# Optical mapping of the 22q11.2DS region reveals complex repeat structures and preferred locations for non-allelic homologous recombination (NAHR)

**DOI:** 10.1038/s41598-020-69134-4

**Published:** 2020-07-22

**Authors:** Steven Pastor, Oanh Tran, Andrea Jin, Danielle Carrado, Benjamin A. Silva, Lahari Uppuluri, Heba Z. Abid, Eleanor Young, T. Blaine Crowley, Alice G. Bailey, Daniel E. McGinn, Donna M. McDonald-McGinn, Elaine H. Zackai, Michael Xie, Deanne Taylor, Bernice E. Morrow, Ming Xiao, Beverly S. Emanuel

**Affiliations:** 1 Department of Biomedical and Health Informatics, The Children’s Hospital of Philadelphia, Philadelphia, PA USA; 2 Division of Human Genetics, The Children’s Hospital of Philadelphia, Philadelphia, PA USA; 3 Department of Pediatrics, Perelman School of Medicine, University of Pennsylvania, Philadelphia, PA USA; 4 Drexel University School of Biomedical Engineering, Science and Health Systems, Philadelphia, PA USA; 5 Institute of Molecular Medicine and Infectious Disease, School of Medicine, Drexel University, Philadelphia, PA USA; 6 Albert Einstein College of Medicine, Bronx, NY USA

**Keywords:** Genome informatics, Genome

## Abstract

The most prevalent microdeletion in humans occurs at 22q11.2, a region rich in chromosome-specific low copy repeats (LCR22s). The structure of this region has defied elucidation due to its size, regional complexity, and haplotype diversity, and is not well represented in the human genome reference. Most individuals with 22q11.2 deletion syndrome (22q11.2DS) carry a de novo hemizygous deletion of ~ 3 Mbp occurring by non-allelic homologous recombination (NAHR) mediated by LCR22s. In this study, optical mapping has been used to elucidate LCR22 structure and variation in 88 individuals in thirty 22q11.2DS families to uncover potential risk factors for germline rearrangements leading to 22q11.2DS offspring. Families were optically mapped to characterize LCR22 structures, NAHR locations, and genomic signatures associated with the deletion. Bioinformatics analyses revealed clear delineations between LCR22 structures in normal and deletion-containing haplotypes. Despite no explicit whole-haplotype predisposing configurations being identified, all NAHR events contain a segmental duplication encompassing *FAM230* gene members suggesting preferred recombination sequences. Analysis of deletion breakpoints indicates that preferred recombinations occur between *FAM230* and specific segmental duplication orientations within LCR22A and LCR22D, ultimately leading to NAHR. This work represents the most comprehensive analysis of 22q11.2DS NAHR events demonstrating completely contiguous LCR22 structures surrounding and within deletion breakpoints.

## Introduction

The 22q11.2 Deletion Syndrome (22q11.2DS) is a congenital malformation disorder and the most frequent microdeletion syndrome in humans^1^. It has a prevalence of about one in every 3,000 live births^1,2^ and one in every 1,000 pregnancies^3^. Significant medical issues present in affected individuals may include: congenital cardiac defects (~ 75%), immune deficiencies, speech/language defects, intellectual disabilities, and a 25–30% risk for developing schizophrenia in adolescence or adulthood^2^. The causative deletion usually occurs as a de novo event in meiosis in one of the parents. Ninety percent of affected individuals have a hemizygous ~ 3 million base pair (Mbp) deletion in chromosome 22q11.2^2,4^. The mechanism responsible for the deletion is non-allelic homologous recombination (NAHR) between surrounding chromosome 22-specific low copy repeats (LCR22s)^5,6^.

There are eight LCR22s, labeled alphabetically as LCRA to LCRH from centromere to telomere on 22q^6^. The LCR22s are comprised of sequence modules of varying lengths containing interspersed genes and pseudogenes. Sequence analysis of these modules reveals a complex organization of duplicated elements. The most frequent ~ 3 Mbp deletion is immediately flanked by LCRA and LCRD, two of the largest LCR22s^2,6,7^. LCRA and LCRD contain a highly homologous (> 99% sequence identity) 160 kbp repeat that is often present in more than one copy in LCRA^7–9^. The combination of large, near-identical segments makes the LCR22s substrates for NAHR, leading to genomic rearrangements. Unfortunately, these characteristics also make the LCR22s difficult to sequence or to reliably identify rearrangement breakpoints in individuals with 22q11.2DS. While significant progress has been made toward elucidating genomic structures and mechanisms involved in leading to the causative 22q11.2 deletion, any predisposing structures and the location of deletion breakpoints remain unknown. Currently, there is no complete model of the 22q11.2DS NAHR mechanism. Such a model would require complete resolution of contiguous LCR22 modules on each chromosome 22 homolog in the parent who transmits the recombination-generated deleted chromosome, along with the resulting 22q11.2 deletion-containing haplotype transmitted to this individual’s proband.

Numerous genomic disorders arise from NAHR of LCRs specific to other chromosomes^10–12^. Previous studies have indicated the majority of microdeletions derived from NAHR are interchromosomal exchanges between homologs in the same, healthy individual^13^. Specifically, in 22q11.2, interchromosomal exchanges represent a higher proportion than intrachromosomal exchanges^13^. Furthermore, predisposition to other genomic disorders, Williams-Beuren syndrome^11^ and 16p12.1 microdeletions^14^, has been linked to copy number variation of subunits within LCRs. Copy number variation in LCR22 modules has yet to be linked to predisposition to 22q11.2DS, as the complex arrangement of the LCR22s has made the identification of any NAHR-driving sequences difficult. Further, the highly variable haplotypes within and between individuals have complicated the analysis^15^.

Current sequencing technologies fail to correctly assemble the 22q11.2 region. This has prevented an in-depth characterization of the haplotypes associated with NAHR and subsequent 22q11.2 deletions. This has further prevented the characterization of sequences or motifs surrounding or within the breakpoints. As a result, it is unknown if NAHR occurs in preferred regions in 22q11.2. Optical mapping is capable of detecting the approximate sizes and locations of structural variants larger than 2 kbp and deciphering highly repetitive regions. Thus, it could be appropriately applied to 22q11.2 to resolve the high-identity, hypervariable complex arrangement of segmental duplications^16^. In 22q11.2, the segmental duplications are significantly larger than any current sequencing technology’s read lengths but can be traversed by optical mapping’s ultra-long molecules.

The purpose of the current study was to produce complete, LCR22-specific haplotypes from optical maps in 22q11.2DS affected individuals and their parents. We characterized parental haplotypes and generated optical map-based NAHR models for the probands’ deletions. We previously reported on the diversity of LCR22s using first generation optical mapping technology and fiber FISH^15^. However, this work contained a limited number of families (seven), which prevented a rigorous comparison of genetic structures associated with NAHR. Further, the population data consisted of incomplete LCR22A haplotypes due to double-strand breaks using the previous iteration of optical mapping nick-labeling technology. Here, the latest optical mapping technology has been used. It employs a labeling scheme that avoids double-strand breaks. This technology has provided a more accurate and contiguous representation of LCR22 structures while confirming previous results on a much larger cohort. The current data identified several more unique LCR22 haplotypes. This generated a larger list of alternative haplotypes in 22q11.2, providing a significant advancement over previous work. Further, since nine genomes overlap with the previous study, this work is able to independently validate haplotypes across two technologies. The present results indicate a preferred local orientation surrounding the NAHR events, with a reference orientation 160 kbp module as the site of recombination and an inversion in LCR22D frequently flanking the rearrangement breakpoint site. Finally, a commonly shared segmental duplication containing *FAM230* gene members is found within each predicted recombination range, suggesting a preferred recombination sequence. To date, this is the largest study of families with completely mapped 22q11.2 regions and elucidation of NAHR events, enabling a robust scaffold for future studies of 22q11.2DS.

## Results

### Long single DNA molecules detect and confirm localized 22q11.2 haplotypes

**Figure 1:**
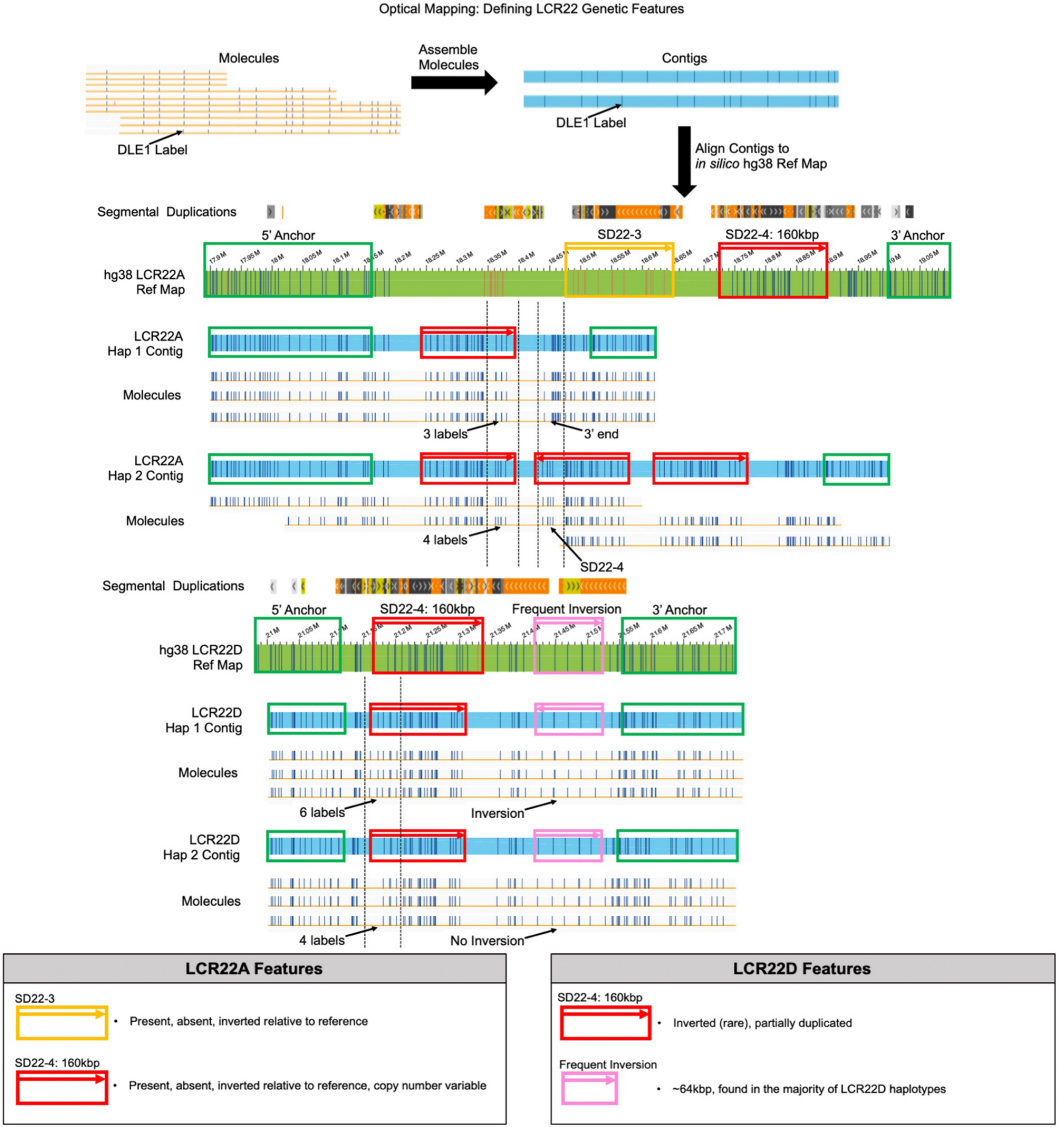
Defining LCR22 features through optical mapping. DLE1-labeled molecules (yellow), > 150 kbp in length, are assembled into contigs (blue), and aligned to the in silico labeled hg38 reference map (green) to obtain individual LCR22A and LCR22D haplotype maps. Molecules comprising haplotypes anchor outside segmental duplications (green boxes) and connect tandem duplicons with unique labels or polymorphic labels. Here, LCR22A has two haplotypes and the top one lacks SD22-3 (mustard) and contains one reference orientation 160 kbp module (red). The second LCR22A haplotype is differentiated from the first by having three 160 kbp modules and again lacks SD22-3. Anchored molecules connecting to the four labels in the 5′-most 160 kbp module differentiate it as a separate haplotype from the first haplotype, which contains three labels at the same reference-based locus. Likewise, the first haplotype contains clear evidence of a contig and its molecules anchoring in the 3′ end whereas the second haplotype continues to the next 160 kbp module. LCR22D also contains two unique haplotypes. Here, the first haplotype contains a 160 kbp module with six 5′ labels and an inversion (pink) and the second haplotype contains a 160 kbp module with four labels at the same reference-based locus and no inversion. Mapping contigs and molecules in all genomes yielded four clearly-defined features, explained in the two boxes. SD22-3 (mustard), SD22-4 (red), and the frequent ~ 64 kbp LCR22D inversion (pink) are named based on^9^ and^15^.

Using DLE1-based optical mapping, localized, contiguous haplotypes in each of the four LCR22s spanning the typically deleted region in chromosome 22q11.2 (LCR22A, LCR22B, LCR22C, LCR22D) were obtained. Given that the families in the dataset consisted of probands with typical LCR22A to LCR22D deletions, we focused on LCR22A and LCR22D. Completely connecting LCR22A to LCR22D in one homolog was not possible in most genomes. This would require heterozygous label differences connected by single DNA molecules across a > 2 Mbp region. To obtain individual, contiguous LCR22A and LCR22D haplotypes, tandem repeats of large segmental duplicons, were linked together using polymorphic labels between paralogous copies of the duplicons (Fig. 1)^9^. Using this approach, two features from LCR22A and two features from LCR22D were identified (Fig. 1; grey boxes), which enabled comparison between haplotypes within and between individuals. The two features in LCR22A were a previously named SD22-3 duplicon and a 160 kbp module. In LCR22D, another 99% identical 160 kbp module and a frequently-observed ~ 64 kbp inversion were the features of interest. Identification of these elements allowed us to clarify features frequently associated with NAHR.

**Figure 2:**
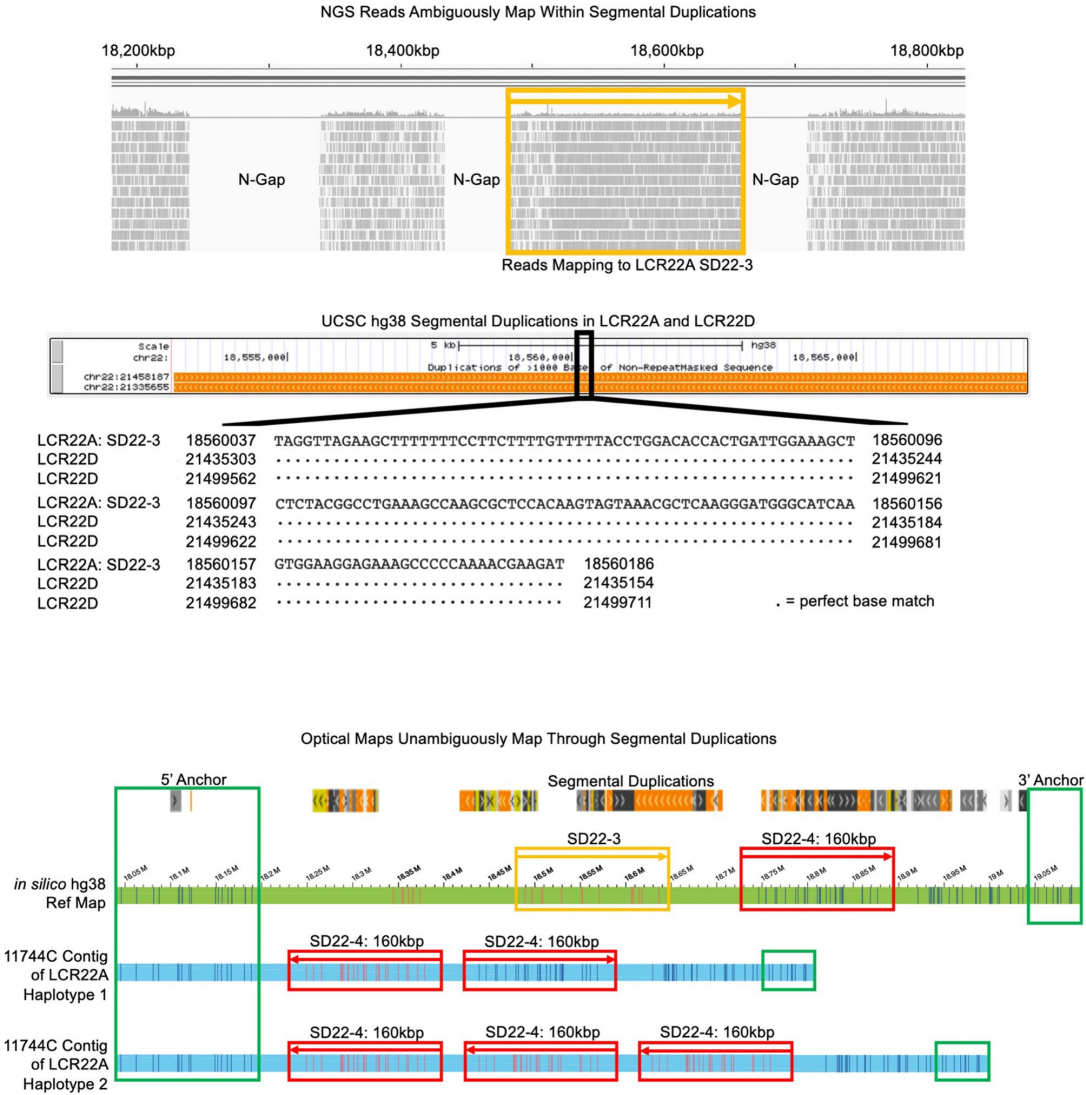
Optical mapping unambiguously reveals the correct structures and haplotypes in 22q11.2. Illumina sequencing reads of the 11744C genome map to the hg38 reference genome 22q11.2 sequence. This includes reads mapping to the SD22-3 segmental duplication (mustard). Upon inspection of SD22-3 in the UCSC Genome Browser’s segmental duplication track, reads mapping to several loci within SD22-3 would also map to other segmental duplications in 22q11.2 with > 98% identity. A small section of SD22-3 is indicated in the figure, where reads mapping from 18,560,037 to 18,560,186 would also map to two LCR22D loci with 100% identity. Optical mapping of the 11744C genome indicates the complete absence of SD22-3 in either of its haplotypes (blue contigs) when aligned to the hg38 reference map (green). One haplotype consists of two copies of SD22-4, a previously characterized 160 kbp element, with inverted and reference orientations. The other haplotype consists of three copies of SD22-4, all inverted relative to the reference. Anchor regions outside the segmental duplications (green) validate correct mapping of contigs, providing clear evidence of these two haplotypes, demonstrating that SD22-3 does not exist as a gross structure in this genome as Illumina short reads incorrectly indicated.

Sequencing technologies cannot properly capture the copy numbers and/or orientations of the segmental duplications within LCR22A and LCR22D. Optical mapping overcomes the obstacle of ambiguous mappings by linking DLE-1 labels from genomic regions lacking segmental duplications (anchor regions), which contain unique label patterns not observed in other genomic loci. In the absence of drastically increasing the permissiveness of molecule and contig alignments (i.e., increasing the allowance of unaligned labels from a molecule or contig to the reference), molecules and contigs could be unambiguously mapped to anchor regions. This provided high-confidence alignments and significant labeled evidence of correct haplotype configurations using well-anchored molecule and contig maps. After defining signature genetic features in LCR22A and LCR22D, we compared our maps containing well-anchored molecules and the above features to those of short-read sequencing data. Using Illumina 150 bp paired-end reads from nine genomes (11744, BM1452, and BM1453 trios) mapped to the hg38 reference, the sequence data were unable to capture the true haplotype configurations that was revealed in the optical mapping data (Fig. 2).

**Figure 3:**
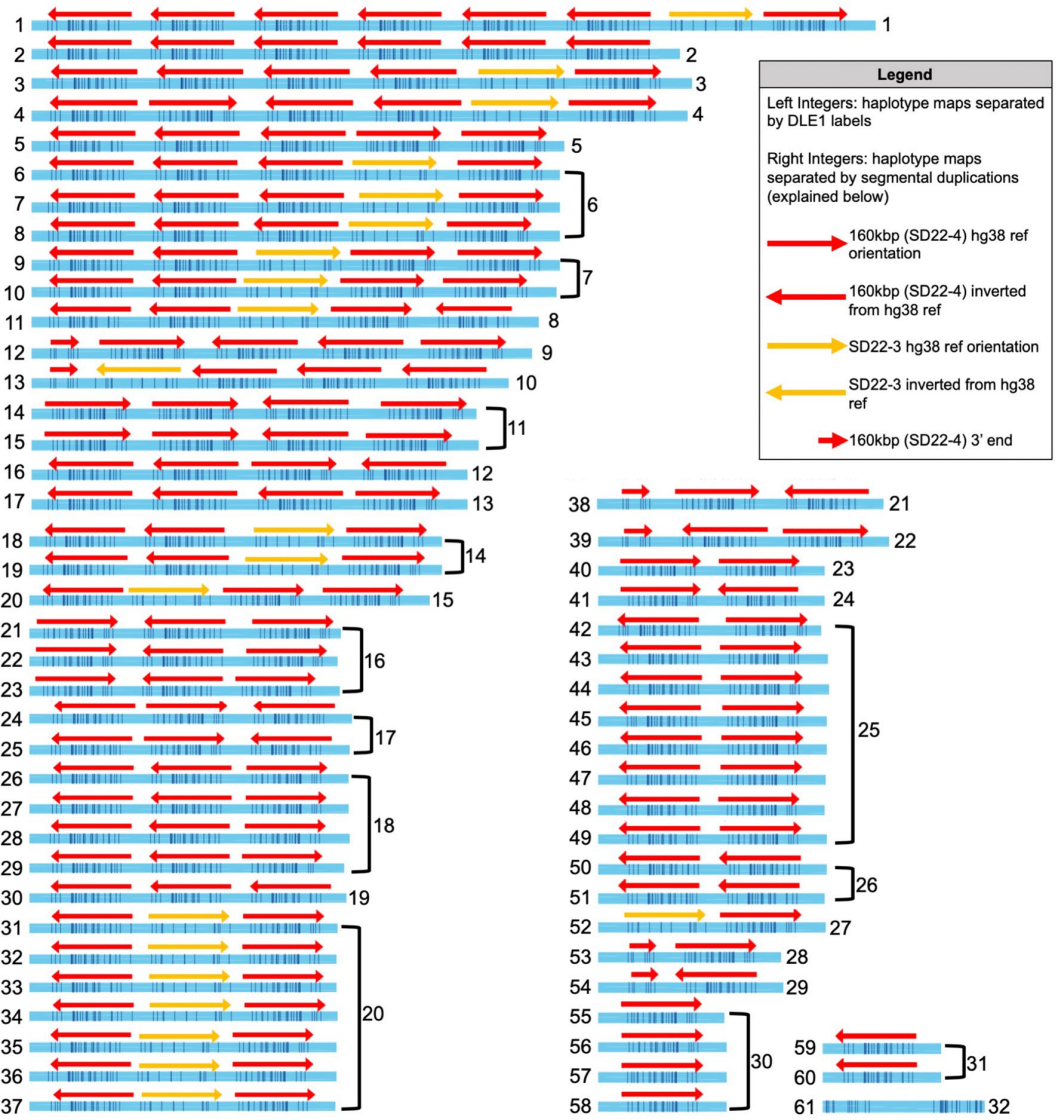
Unique LCR22A structures across parental genomes. LCR22A haplotypes were grouped based on the two defined segmental duplication features (see Fig. 1) and DLE-1 label patterns. The left-most integers of each haplotype contig map (blue) indicate maps grouped by DLE-1 labels (61 groups) and the right-most integers indicate maps grouped by the two segmental duplication features (32 groups). The 160 kbp modules (red arrows) and SD22-3 modules (mustard arrows) were present, absent, or copy number variable in any orientation relative to the hg38 reference map. The partial 160 kbp modules (smaller red arrows) were only present in single copies and in reference orientation.

In LCR22A, high variability in haplotype maps, as compared to the hg38 reference, was identified, validating previous results (Fig. 3)^15^. Much of the variability was derived from the copy number changes of a 160 kbp module^9^, of which up to seven copies in a single haplotype have been found (Fig. 3, LCR22A haplotype ID 1). When considering only segmental duplication variability compared to the hg38 reference, 32 unique LCR22A haplotypes across all genomes were identified (Fig. 3, right-most integers). The variability of LCR22A was further characterized by the presence of only 10 homozygous parental genomes out of 57 total parental genomes. SD22-3 (Fig. 3 mustard arrows) was present in 27 parental haplotypes across 25 genomes and there was only one instance of an inverted copy relative to the reference. The most frequent parental haplotype contained a single, reference orientation 160 kbp module (33/114 haplotypes). Interestingly, 18 of the 32 haplotypes were found only once. Instances of partial 160 kbp modules, corresponding to the last 50 kbp of the entire module were also found. This partial 160 kbp module was only present as the first segmental duplicon in haplotypes such that there were no other SD22-3 or 160 kbp modules 5′ of it. Given the presence of polymorphic labels between individual segmental duplicons, LCR22A maps were also stratified by label differences. When considering haplotypes grouped by DLE-1 labels, 61 unique LCR22A haplotype maps were obtained (Fig. 3, left-most integers). Grouping by labels reduced the homozygous parental genomes to only one genome (11280B). The most polymorphic duplicon with respect to labels was the 160 kbp module with little variation observed in SD22-3 and no variation in the partial 160 kbp module.

**Figure 4:**
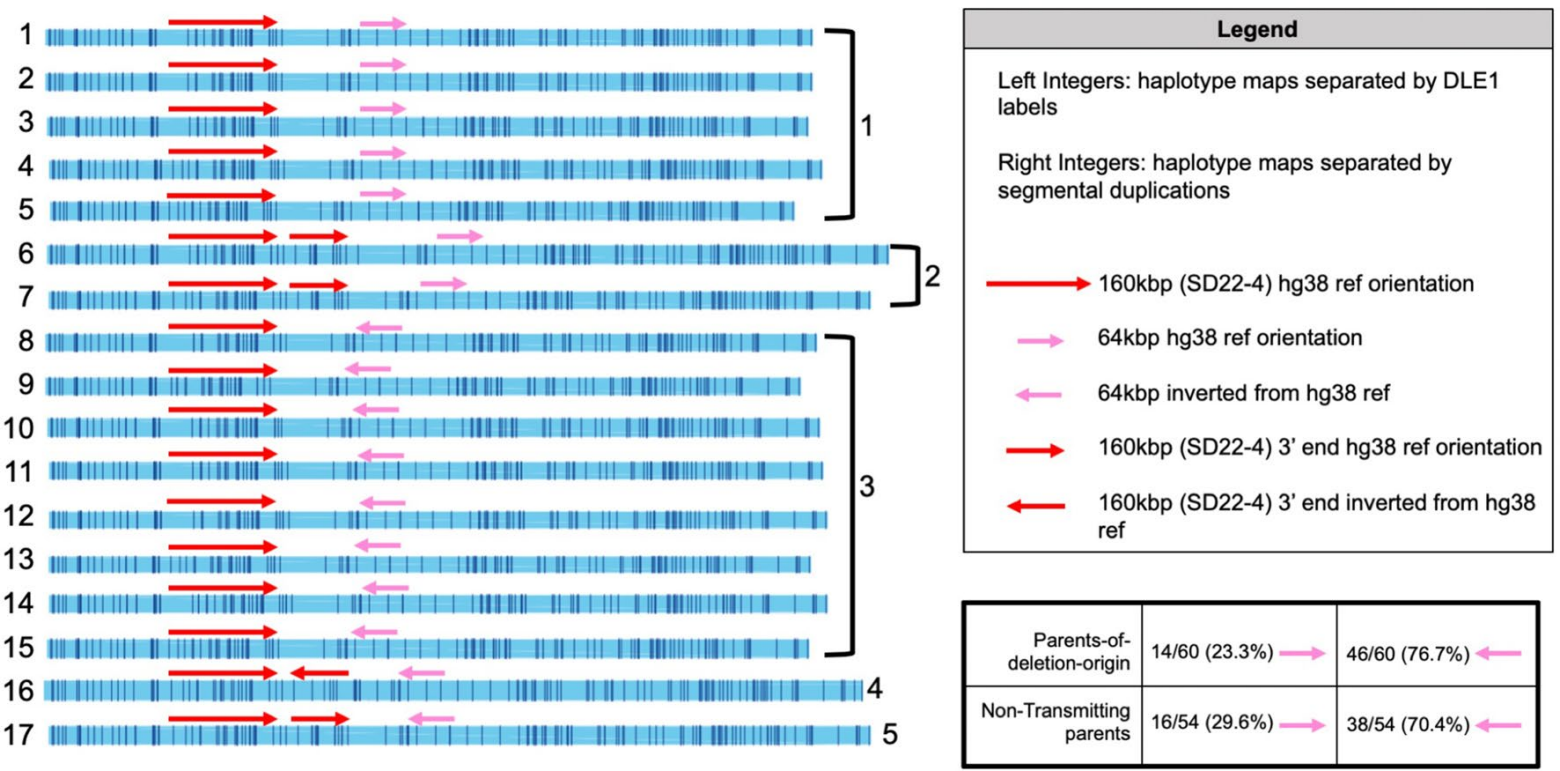
Unique LCR22D structures across parental genomes. Optical maps reveal a relatively stable LCR22D configuration across haplotypes and genomes with very few changes to the 160 kbp module (red arrows) but a frequent inversion polymorphism (pink arrows). Left-most integers indicate the maps grouped by DLE-1 labels and right-most integers are maps grouped by the previously defined segmental duplication features. There are also instances of partial 160 kbp duplications (left-most integer labels 6, 7, 16, and 17). The majority of LCR22D parental haplotypes contain the inversion and no significant difference in the frequency of the inversion between parents-of-deletion-origin LCR22D haplotypes as compared to non-transmitting parents was observed (bottom-right box).

Using the same procedure as in LCR22A, we defined two major features in LCR22D haplotypes. This process defined five unique LCR22D haplotypes across all parental genomes (Fig. 4 right-most integers). The most prominent feature was a ~ 64 kbp inversion (hg38 DLE-1 label positions 21,424,743–21,510,142) and was found to be in the majority of our haplotypes (Fig. 4 pink arrows). The inversion was found in 84/114 (73.7%) parental LCR22D haplotypes, perhaps signifying that the reference configuration represents the minor allele. When comparing the proportion of LCR22D haplotypes with and without the inversion in parents-of-deletion-origin to NAHR-negative parents, no significant disparity of the inversion was found between these parental groups. This finding, combined with knowledge of a high frequency of the inverted allele in the general population, may not signify the LCR22D inversion as a predisposing factor to NAHR leading to the 22q11.2 deletion. Aside from the inversion, other configurations of LCR22D contained a reference orientation or inverted duplication of a portion of the 160 kbp module (~ 21.26–21.32 M). Unlike in LCR22A, an intact inverted 160 kbp module was not observed in LCR22D. When considering DLE-1 labels, 17 unique LCR22D haplotypes were defined (Fig. 4 left-most integers).

### NAHR-specific haplotypes

**Figure 5:**
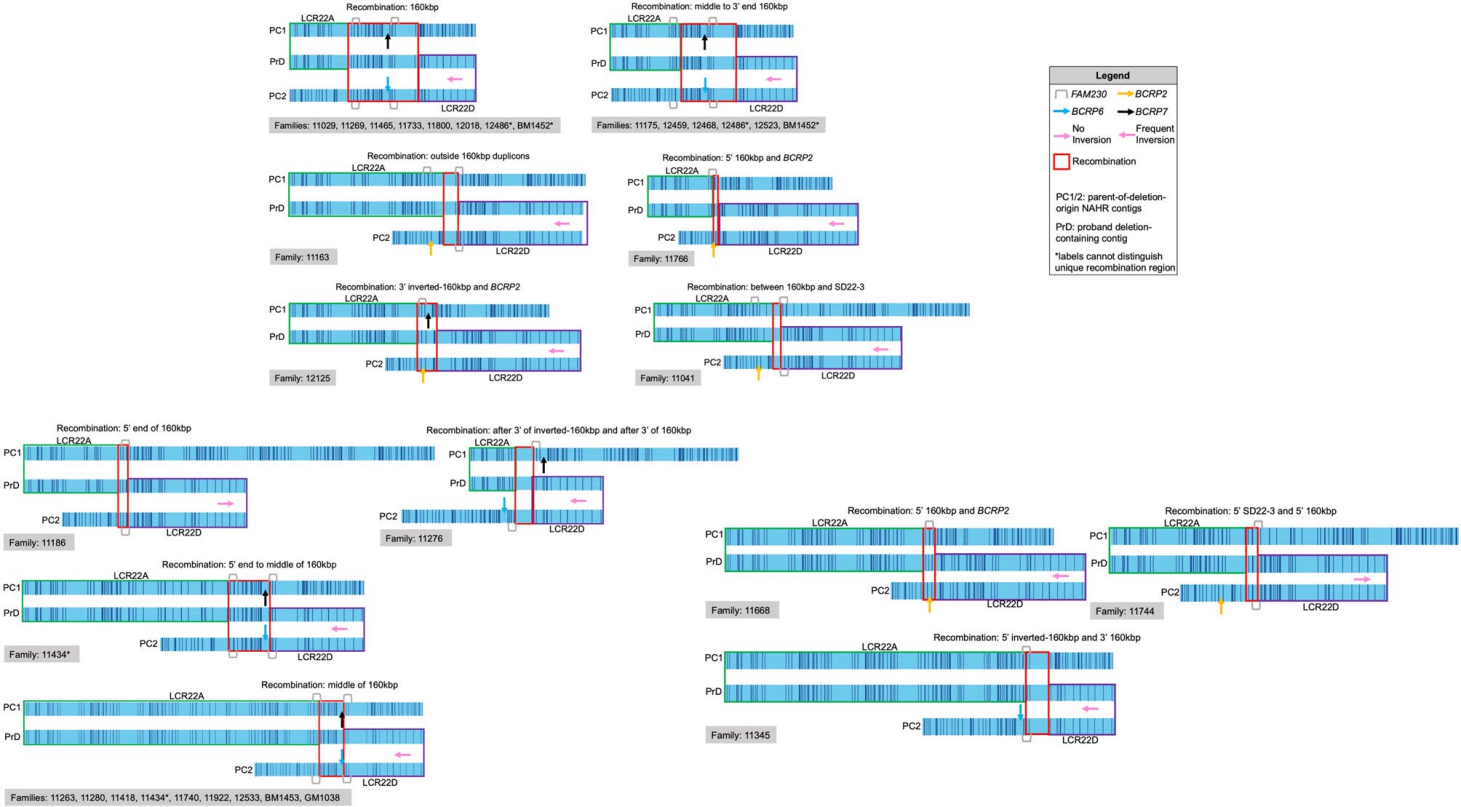
Thirteen unique estimated NAHR events across 30 families. In each event, a parental LCR22A contig (blue) is the top-most contig and the opposing parental LCR22D contig is at the bottom. The middle contig is a proband’s deletion-containing contig from the families listed below in the gray box. Matched labels between parental LCR22A (green boxes) and the proband contigs signify unambiguous LCR22A before the site of NAHR. Matched labels between parental LCR22D (purple boxes) and the proband contigs signify unambiguous LCR22D after the site of NAHR. Red boxes signify the estimated ambiguous site of recombination, denoted by shared labels across parental LCR22A and LCR22D contigs. All NAHR events overlapped with *FAM230* sequences. Estimated NAHR ranges are not to scale.

Initially, the hypothesis that a structure or specific configuration might be associated with NAHR leading to the 22q11.2 deletion was considered. If so, this might indicate a predisposing configuration in parents-of-deletion-origin. Considering LCR22A, the most frequent recombining haplotype contained a single reference orientation 160 kbp module (Fig. 3 right-most ID 30 (four maps)). This haplotype occurred 21 times in parents-of-deletion-origin and 13 of these were found in recombining haplotypes. This haplotype was also found in 11 non-transmitting parents and four of these were inherited in probands. When considering label distributions, this observation holds up. Of the 13 LCR22A NAHR-associated haplotypes, six were unique to parents-of-deletion-origin while seven were also present in NAHR-negative parents. Since parents-of-deletion origin and NAHR-negative parents had this haplotype, the entire haplotype cannot be solely associated with NAHR and may not be a predisposing haplotype. When all parent-of-deletion-origin LCR22A haplotype frequencies were compared to NAHR-negative parents, no significant differences were observed (Fisher’s exact test, *p* = 0.4321). Of note however, was that the majority of NAHR events occur inside reference orientation 160 kbp modules located in both LCR22A and LCR22D (24/31 NAHR events, Fig. 5). Thus, while specific LCR22A haplotypes were not found to be solely associated with NAHR, the reference orientation 160 kbp modules were more frequently associated with NAHR events.

Similar to LCR22A, no LCR22D haplotypes were observed to be specific to parents-of-deletion-origin. The majority of deletion-containing haplotypes in probands contained the ~ 64 kbp inversion (26/31 haplotypes, 83.9%). Despite the larger proportion, a significant difference between the proportion of NAHR-negative parents’ LCR22D inversions to deletion-containing haplotypes was not observed (Fisher’s exact test, *p* = 0.1990). To determine whether the inversion was a possible predisposing factor, the proportion of inversion haplotypes in the parents-of-deletion-origin and the other parent were compared (Fig. 4). No significant difference in the proportion of LCR22D inversions in parents-of-deletion-origin (46/60 haplotypes, 76.7%) compared to the NAHR-negative parents (38/54 haplotypes, 70.4%) was found (Fisher’s exact test, *p* = 0.5248). Finally, all parent-of-deletion-origin LCR22D haplotype frequencies were compared to non-transmitting parents and no significant differences between the two groups were observed (Fisher’s exact test, *p* = 0.3465).

### Preferred locations of NAHR via optical maps

Paralogous label polymorphisms between segmental duplications (Figs. 1 and 2) enabled the identification of specific LCR22A (Fig. 3) and LCR22D (Fig. 4) regions involved in NAHR (Fig. 5). Specifically, label differences in the 5′ and 3′ ends of 160 kbp modules were observed, as noted previously^15^. When comparing the reference sequences encompassing the label polymorphisms, the additional label present in the reference LCR22D position 21,300,467 was due to a G- > C base change. In other words, at the comparable LCRA position, the 6 bp motif is “**G**TTAAG” whereas the LCRD motif is “**C**TTAAG.” Likewise, LCR22A position 18,746,350 contains a DLE-1 label where the comparable LCR22D position lacks one. Again, a single base change generated a DLE-1 motif in LCR22A (“CTT**A**AG”) and prevented one in LCR22D (“CTT**C**AG”), albeit in a different position within the motif. This may indicate the polymorphic labels as paralogous SNPs although we cannot be certain since base-level precision is not possible.

As small as a ~ 20–30 kbp region of LCR22A-LCR22D recombination site was observed (Fig. 5, family 11041). The majority of recombinations took place within the 160 kbp LCR22A-LCR22D modules (24/31 deletion-containing haplotypes, Fig. 5). Nine of these recombinations were further refined to the middle ~ 90–100 kbp of 160 kbp modules. Only one NAHR event was found to occur between an inverted 160 kbp element in LCR22A and the ~ 21.08–21.15 M region, which is 5′ of the 160 kbp element in LCR22D (Fig. 5, family 12125). Previously, all NAHR sites were localized to 160 kbp modules^15^. However, in this study, seven instances of recombinations outside 160 kbp modules were observed. These events represented one of three categories: (1) immediately flanking 160 kbp modules (two families), (2) between an LCR22A 160 kbp module and LCR22D Breakpoint Cluster Region Pseudogene 2 (*BCRP2*, three families), and (3) between an LCR22A SD22-3 module and LCR22D 160 kbp module (two families).

### Preferred locations of NAHR via sequence

To gain insight into the sequences present in recombination loci, their hg38 reference sequences were compared. The recombination events shared sequence, in the form of segmental duplications, with several loci across chromosomes 1, 2, 5, 6, 13, 16, 20, 21, and 22. Further, many of the recombination loci sequences were found in other LCR22s. Since the sites of recombination in typical deletions were the object of study, we focused on the union of loci in LCR22A and LCR22D. Within 24 families’ unambiguous NAHR loci, *BCRPs* were found. It was previously hypothesized that *BCRPs* may be the site of recombination in typical deletions^9^. In the deletion loci here, *BCRPs* 2, 6, and 7 were within recombination regions from LCR22D (21,103,016–21,122,286 or 21,290,760–21,294,586) and LCR22A (18,855,621–18,858,640). Given this, it was found that *BCRPs* at the site of recombination was not required for a NAHR event to take place, since 6 families had recombinations without *BCRP*.

Despite the absence of *BCRP*-based recombinations in all occurrences, sequences in all recombination events are shared among one another. It is possible that there may be several recombination hotspots within these multi-kbp sequences or that the *BCRPs* may not be the sites of exact recombination. Resolution limitations of optical mapping do not enable an exact determination of NAHR ranges and therefore the explicit determination if all events in Fig. 5 share the exact same sequence was not possible. The estimated ambiguous NAHR sites, however, reveal that all NAHR events in Fig. 5 contain or overlap with either the *FAM230A* (18,422,244–18,500,594) or *FAM230B* (21,169,946–21,182,974) lncRNAs, as annotated by NCBI RefSeq in hg38. Thus, the preferred site of recombination among all of the NAHR events contained *FAM230* lncRNAs.

Overall, these data suggest a frequent recombination location and specific LCR22 configurations surrounding the deletion breakpoints. Most LCR22 configurations in the NAHR events occurred between reference orientation 160 kbp modules flanked distally by LCR22D inversions. The exact sites of recombination events share a common segmental duplication of the *FAM230* members and may not require *BCRP* sequences.

## Discussion

To date, this is the most complete elucidation of 22q11.2 LCR22A and LCR22D haplotypes and NAHR events in families consisting of healthy parents and 22q11.2DS-affected probands. The development of a localized de novo assembly approach combining the knowledge of inherited polymorphic labels enabled the determination of structures frequently involved in NAHR. Our ultra-long DNA molecules permitted the traversal of tandem modules within segmental duplications where sequencing approaches typically collapse. The added advantage of optical mapping is observed in its high-throughput nature^16^. Whole human genomes can be mapped in less than one week enabling efficient use of time to determine the long-range structures of LCR22s.

The previously described variability in LCR22A (18.1–19.0 M hg38 reference coordinates) (Fig. 3) and a characteristic ~ 64 kbp inversion polymorphism in most LCR22D haplotypes (21.0–21.7 M hg38 reference coordinates) was observed (Fig. 4)^15^. Due to the difference in probe density/design in the previously reported fiber FISH experiments in^15^ and the label distributions of DLE-1, some of the LCR22A haplotypes were not detectable in the same way. Considering only the haplotypes that contain fiber FISH probes with DLE-1 labels and removing consideration of possible discrepancies in smaller elements defined by fiber FISH probes, 19 new LCR22A haplotypes were observed in the current study. It has been suggested that allelic homologous recombination between LCR22A modules may play a role in the evolution and diversity of this locus^15^. In this study, no direct evidence of expansion or contraction of LCR22A segmental duplicons in related individuals over generations was observed, although this was not specifically studied in this cohort of subjects.

The density of DLE-1 labels (15–17 labels/100 kbp on average) in conjunction with length-based filtering of molecule maps enabled the confirmation of unambiguous haplotypes in individuals (Figs. 1, 2, 3, and 4). This permitted the determination of parental haplotypes undergoing NAHR leading to the 22q11.2 deletions (Fig. 5). However, based on the experimental design herein, it was not possible to determine with certainty whether the recombination events were the result of inter- or intrachromosomal NAHR.

An added benefit of the DLE-1 labeling density and using families was the determination of specific labels inherited in deletion-containing haplotypes. This allowed for further refinement of highly identical recombination loci found in NAHR events (Fig. 5). The majority of NAHR events took place between LCR22A and LCR22D 160 kbp elements. These modules share high sequence identities (> 99% overall). Despite this, the 5′ ends (first ~ 40 kbp) and 3′ ends (last ~ 30 kbp) of the modules frequently contained polymorphic labels between paralogs. Thus, the recombination loci of eight NAHR events were determined in the ~ 90–100 kbp segment of the modules.

Our initial hypothesis was that specific haplotypes might predominate in the parent-of-deletion-origin. If this were true, the ideal goal would be to predict the likelihood of a deletion event before conception based on parental haplotypes. However, when comparing LCR22A haplotypes between parents, there are instances of completely shared haplotypes. In the most common NAHR-associated LCR22A haplotype, a single reference orientation 160 kbp element is shared with 11 NAHR-negative parents. In addition, there are no LCR22D haplotypes unique to either parental group. To date, the majority of haplotypes in any mapped population contain the ~ 64 kbp inversion (Fig. 2). While this inversion was present in 83.9% of deletion-containing haplotypes, it may not be a prerequisite for NAHR. Previous studies have found no evidence of parental inversion polymorphisms in sequences flanking the 22q11.2 deletion^17^. Because of the variability between haplotypes, small sample size, and shared optical map-based features between parents, it is not yet possible to predict a pre-conceptual parent-of-deletion-origin.

Despite the lack of haplotypes unique to parents-of-deletion-origin, frequently associated structures were observed which may increase the possibility of NAHR occurrences. A reference orientation 160 kbp module in LCR22A and LCR22D flanked by an LCR22D inversion was the most frequent recombining locus. Further, the less complex LCR22A haplotypes (i.e., one reference orientation 160 kbp module) were the highest proportion of NAHR-associated haplotypes, although this was also the highest proportion haplotype in non-transmitting parents. One may think a more complex LCR22A provides additional opportunities for recombination with LCR22D but this also means more opportunities for allelic recombination between homologous LCR22A haplotypes. It may be that less complex LCR22A structures, combined with LCR22D inversion, lead to more frequent recombination events. Comparisons to African and African American individuals may help explain this, as they have proportionally fewer LCR22D inversions^15^ and perhaps their LCR22A haplotypes are more complex. Previous work using the older nick-labeling technology^15^ could not contiguously resolve LCR22A, whereas African American samples studied with the latest DLE-1 technology might shed light on this phenomenon.

The sharing of map-based structures between parents-of-deletion-origin and NAHR-negative parents does not mean there are no features specific to NAHR events or which predispose them. Sequences below the resolution of our maps may contain SNPs or indels, which bring together otherwise different modules into contact leading to recombination. Further, microhomology-mediated mechanisms have been implicated in other microdeletions and genomic rearrangements and regions may be flanked by *Alu*, LINE, or HERV elements^10,18,19^. In 22q11.2, an *Alu*-based polymorphism in LCR22E in close proximity of an LCR22D-LCR22E deletion breakpoint may have provided genomic instability required for a previously published NAHR event to occur^20^. The polymorphism provided matching orientation *AluY* sequences in both LCR22D and LCR22E regions near the aberrant recombination where typically these regions would lack the shared, high-identity sequences^20^. To explore this further, sequencing the breakpoints in atypical and nested deletions is currently under way which may reveal the sequences involved in recombination events.

While most NAHR events occurred between reference orientation 160 kbp modules (Fig. 5), one recombination involved an inverted 160 kbp module. The maternal LCR22A haplotype in sample 12125B recombined at the 3′ labels of an inverted 160 kbp module with sequence in LCR22D. Given this study’s results and previously mapped recombination events^15^, this may represent a rare NAHR occurrence. As shown in the 12125 trio NAHR figure (see Supplementary Fig. S1 online), there were smaller modules of shared surrounding sequences between LCR22A and LCR22D parent-of-deletion-origin homologs. The decreased incidence of NAHR events directly involving inverted 160 kbp modules may be described by two observations. A paucity of inverted 160 kbp modules in LCR22D has been observed, preventing direct recombination between these modules and inverted 160 kbps modules in LCR22A. Further, as indicated in the 12125 trio, inverted 160 kbp modules in LCR22A may recombine with smaller modules in LCR22D haplotypes lacking inverted 160 kbp modules. Since this NAHR event occurs between a smaller high identity module than most of the observed NAHR events, it is possible the shorter length of shared sequence makes aberrant recombinations occur less frequently.

Since NAHR events occurred between different loci, the reference sequence was analyzed for any shared motifs. According to RefSeq Genes and Pseudogenes, the 18,855,621–18,858,640, 21,103,016–21,121,784, and 21,290,760–21,294,586 positions contain Breakpoint Cluster Region Pseudogenes (BCRP) 7, 2, and 6, respectively. BCRPs are the pseudogene equivalent of BCR, the chromosome 22 junction of the 9;22 translocation of CML^21^. BCRPs 7 and 6 reside within the 160 kbp modules of LCR22A and LCR22D, respectively, and may be in or near the site of frequent NAHR events (Fig. 5) as one study hypothesized^9^. Several trios used in that study were mapped with fiber FISH^15^ and are included in the present study (BM1452 and BM1453). BM1453.001 is the proband with the most precisely identified breakpoint region according to sequence data^9^. A potential breakpoint residing in or near the BCRP2, BCRP6, or BCRP7 sequences^15^ was confirmed. The observation of these loci within the most frequently occurring recombination region suggested this might be the site of numerous recombinations. However, they are not present in all of our NAHR events (Fig. 5). Events between one or no BCRPs were observed which have two implications: (1) BCRPs are not the universal site of recombination or (2) both BCRPs and other highly identical sequences are the sites of recombination. Since NAHR can occur between highly similar sequences, BCRPs appear not be a prerequisite or they may offer an explicit advantage to surrounding, equally identical sequences on opposing homologs^22^.

As indicated in Fig. 5, despite positional differences, other DNA motifs in these events share sequences within recombination loci between and outside 160 kbp modules (*FAM230A* and *FAM230B*). This segmental duplicon was previously hypothesized to be directly associated with NAHR events, albeit within only seven families^15^. This finding was confirmed in our 30 families. It is possible that recombination events outside 160 kbp modules occur less frequently due to the shorter length of highly-identical sequences on misaligned LCR22A and LCR22D loci. This will perhaps be answered when additional families of differing ethnicities are mapped.

Aside from long stretches of high sequence identity, it is currently unknown why the recombinations observed here occurred within a highly identical sequence motif across different loci. This could be because the pairing of homologs in meiosis between nearly identical sequences occurs at these locations with breaks before and after. It is interesting to note that in 160 kbp modules, label polymorphisms in the 3′ end flank the most frequently recombining locus. This may explain why despite differences in sequence surrounding the recombination sites that create the label polymorphisms, a recombination still occurs. Conversely, these surrounding sequences still have very few differences. Some instances of label polymorphisms arise due to SNPs. This may be because even though they share sequence and can in principal recombine, the surrounding sequences may not be as identical (i.e., segmental duplication modules may be in a different order). Previous studies found SD length correlated to recombination and this may be the case here as well^10,23^. Overall, length and identity of SDs may explain why LCR22A and LCR22D recombinations define the typically deleted region, as other deletions (nested, distal, and especially atypicals) have less surrounding sequence identity.

Across all families, there were 19 female parents-of-deletion-origin and 11 male parents-of-deletion-origin. This gender discrepancy has been noted previously, where an enhanced maternal origin of 22q11.2DS was observed^12^. Specifically, the female recombination rate was approximately 1.6–1.7 times greater than that for males. Our study agrees with this finding, as we identified a female recombination rate of ~ 1.7 times greater than males.

While the phenotypes associated with probands was not analyzed, the 22q11.2 deletion could potentially alter phenotype based on the elements remaining in the LCR22s after the deletion occurs. The variability in LCR22A genomic structures in deletion-containing haplotypes and the variability of the LCR22D inversion may be associated with phenotypic variability. However, our study contained a family with identical twins (11280). One proband, 11280A, contained a congenital heart defect, while the other, 11280D, did not. As expected, the probands contained identical optical maps. Based on this observation, phenotypic variability may be comprised of multiple factors; genomic components as well as stochastic mechanisms (gene-environment relations, in utero environment, etc.).

Previous studies have indicated decreased proportion of the ~ 64 kbp LCR22D inversion in African and African American populations compared to Europeans, East and South Asians, and South Americans^15^. Prior studies of 22q11.2DS at our institution have indicated an under-representation of African Americans in the local 22q11.2DS patient group^24^. If the LCR22D inversion appears in African American parents-of-deletion origin with increased frequency as compared to non-NAHR parents or population-based African Americans, it could potentially help to explain this decrease. Further, the complex structures of LCR22s may play a role, by virtue of increased frequencies of inverted 160 kbp modules in LCR22A and/or variability in segmental duplications in LCR22D. Ultimately, additional studies of 22q11.2DS in various ethnicities may help unravel the role of LCR22s, perhaps illuminating the mechanism(s) driving this common deletion.

In this study, optical mapping was used to define the structure and describe the variation of LCR22s in 88 people across 30 22q11.2DS families. Using these maps, specific NAHR locations and genomic signatures associated with the deletion were observed. Despite the fact that no whole-haplotype predisposing configurations were identified, optical mapping results suggested preferred recombination sequences, as all NAHR events contained a segmental duplication encompassing *FAM230* gene members. Upon analyzing the putative deletion breakpoints in all families, the preferred recombination occurs between *FAM230* gene members and frequently-observed segmental duplication orientations within LCR22A and LCR22D, ultimately leading to 22q11.2DS**.**

## Methods

### Sample composition, collection, and consent

Subjects for the study were primarily families consisting of two healthy parents and an affected proband with a de novo ~ 3 Mbp deletion in 22q11.2. The study cohort was comprised of 26 trios, one quad (identical twin probands), and three duos where the parent available was the parent of deletion origin. Patients and their non-deleted parents were tested for the presence or absence of the deletion using either a FISH assay with N25 probes (Abbot Molecular, Abbot Park, Illinois, USA) or the MLPA SALSA P250 DiGeorge diagnostic probe kit (MRC-Holland). Informed consent was obtained from all participants and/or their legal guardians. These individuals and/or their legal guardians provided written consent for their EBV cell lines and DNA to be used for research purposes. The study was approved by the Children’s Hospital of Philadelphia under the Institutional Review Board (IRB) protocol 07-005352. All experiments were performed in accordance with the relevant IRB guidelines and regulations.

### Parent-of-deletion-origin detection

Eighteen short tandem repeat polymorphisms (STRPs) or microsatellite markers within the deleted region (see Supplementary Table S1 online) were used to determine the parent-of-deletion-origin. Fluorescently labeled PCR products were analyzed on an ABI 3730 instrument in the Nucleic Acid/Protein core facility at the Children’s Hospital of Philadelphia. The GeneMarker software (Version 2.0) was used to analyze the size and relative intensity of each product. A minimum of three informative markers per trio were required to assign parent of origin. An informative marker would typically consist of a proband matching one parent, but not the other.

### High molecular weight (HMW) DNA isolation for optical mapping

High Molecular weight DNA isolation was performed based on manufacturer’s protocol (https://bionanogenomics.com/wp-content/uploads/2018/02/30026-Bionano-Prep-Cell-Culture-DNA-Isolation-Protocol.pdf). Briefly, lymphoblast cultures were established for eventual HMW isolation. Lymphoblasts were placed into gel plugs, melted, and purified through drop dialysis. DNA samples with a concentration between 36 and 150 ng/μL and high viscosity were used in the following DNA labeling experiment.

### DLE-1 labeling and chip loading

Purified HMW DNA was labeled according to Bionano Prep Direct Label and Stain (DLS) Protocol (Bionano, #30206, Rev. D). Briefly, HMW DNA was pipetted with labeling master mix and incubated for 2 h in a thermocycler. This mixture was purified with proteinase K and further cleaned on a DLS membrane (supplied by Bionano). Labeled DNA was mixed with staining master mix. Stained, labeled DNA was spun down, homogenized, and stored overnight. Labeled and stained samples with a concentration between 4 and 12 ng/μL were loaded onto a dual flowcell Saphyr chip (Bionano). The chip was covered with a Saphyr clip and placed on the Bionano Saphyr instrument. The Saphyr instrument imaged fluorescently labeled DNA molecules. Generally, after 24–36 h, a flowcell on a Saphyr chip generated 320-480Gbp of data.

### Data pre-processing

Tab-separated BNX files containing molecule length, label quality scores, and label locations were output from the Bionano Saphyr optical mapping platform. BNX files were filtered for as long a length while maintaining the manufacturer’s recommended 320Gbp of total molecule data. Long molecules were required to uniquely span tandem duplicon modules, some ranging > 160 kbp, which is longer than the manufacturer’s default 150 kbp cutoff for molecules. Molecule qualities were assessed by the Bionano Access Molecule Quality Reports and compared to manufacturer’s recommended values (https://bionanogenomics.com/wp-content/uploads/2018/04/30223-Saphyr-Molecule-Quality-Report-Guidelines.pdf). Additional data were collected in samples not meeting these values.

### Localized de novo assembly pipeline

Human genome reference build 38 was in silico nicked with the direct labeling enzyme (DLE-1). Molecules from BNX files were mapped with higher confidence than default to each chromosome, except chromosome 22, using Bionano Genomics’ RefAligner (derived from Bionano Solve versions 3.3 and 3.4) program (notable parameters: -M 3 3 -FP 0.918057 -FN 0.099062 -sf 0.233588 -sd 0.090609 -S 0 -minlen 200 -minsites 15 -T 1e-25 -res 3.5 -resSD 0.7 -Mfast 0 -biaswt 0 -A 5 -BestRef 0 -nosplit 2 -outlier 1e-7 -endoutlier 1e-7 -RAmem 3 30 -hashgen 5 4 2.4 1.4 0.05 5.0 1 1 1 -hash -hashdelta 14 10 24 -hashoffset 1 -hashrange 1 -hashGC 300 -hashT2 1 -hashkeys 1 -hashMultiMatch 30 10). Chromosome 22 was not included in these alignments because of the high variability in LCR22 regions, potentially leaving out molecules otherwise mapping to legitimate haplotypes. We increased the default molecule mapping p-value from RefAligner to 1e-25 to ensure only higher confidence non-chromosome 22 mappings occurred and to ensure polymorphic chromosome 22-specific molecules did not map to another chromosome. The original BNX file was filtered for molecules mapping to all non-chromosome 22 maps using the RefAligner skipidf command.

Filtered BNX files were de novo assembled using default parameters in Bionano Solve v3.3 and v3.4. Molecules and Assembled consensus maps (CMAPs) were aligned to the in silico nicked hg38 reference map.

### Defining anchor and ambiguous regions in the hg38 reference map

We used data from 6 genomes (families BM1452 and BM1453) optically mapped in this study, which also contained fiber-FISH maps from previously^15^, to define anchor and ambiguous regions in LCR22A and LCR22D of the hg38 reference map. Further, since two independent groups manually assembled and validated the haplotypes in these genomes with orthologous technologies, we used these genomes as a basis to generate the methods in haplotype validation, which we describe further below.

Based on mapping results of the aforementioned 6 genomes, the reference coordinates of LCR22A and LCR22D were demarcated into frequently rearranging repetitive modules and less variable (based on DLE-1 label distribution) genomic regions. The less variable regions were chosen as anchors flanking the segmental duplications in LCR22A and LCR22D and lacked segmental duplications themselves. Specifically, the anchor was defined as 18–18.15 Mbp for 5′ LCR22A, 19.035–19.15 Mbp for 3′ LCR22A, 21–21.11 Mbp for 5′ LCR22D, and 21.565–21.7 Mbp for 3′ LCR22D. Each region provided at least 12 labels and over 100 kbp in length. Molecules mapped to these regions could not map elsewhere without drastically changing the False Positive (-FP), False Negative (-FN), minimum mapped sites (-A), and minimum mapped length (-minlen) from the RefAligner parameters defined above.

### Paralogous label polymorphisms validate assembled haplotype maps with single DNA molecules

Label distribution differences between paralogous 160 kbp mapped elements enabled the chaining of molecules from anchor regions and across tandem 160 kbp elements. As previously defined, 160 kbp modules frequently contained polymorphic labels at the 5′ and 3′ ends^15^. These labels further enabled the segregation of 160 kbp modules within the same haplotype, between haplotypes, and between LCR22A and LCR22D.

Assembled CMAPs were mapped to the hg38 reference genome in the de novo assembly pipeline. CMAP labels mapped to reference anchor labels were defined as CMAP anchors. Two unique CMAPs mapped to the same reference region were defined as individual haplotype maps. Molecules confirmed haplotypes by mapping to anchor CMAP labels and into CMAP labels mapping to segmental duplications, also defined by the reference coordinates. Molecules must map with a summed p-value of 1e-11 (The “T” parameter in RefAligner). This p-value is the recommended mapping threshold by Bionano Genomics. When haplotypes in CMAPs contained the same label pattern in segmental duplications, molecules must anchor and span to the nearest unique polymorphic label distribution. Otherwise, the molecules were retained but marked as ambiguously mapped. In the cases of unique haplotype maps, molecules were re-aligned to both CMAPs and if the molecules could not map to both the anchor and the polymorphic labels, then they were retained as confirming their original mapping (using the same p-value threshold). All haplotypes and their unambiguously mapped molecules were manually confirmed using the Bionano Access visualization software v 1.4.1 (June 21, 2019 build). The same process was used for chaining long haplotype maps (e.g., > 1 160 kbp modules in tandem). In these cases, molecules nested within segmental duplications must uniquely map to polymorphic labels. Only when single DNA molecules uniquely mapped to a single, assembled haplotype Consensus Map (CMAP) and crossed from 5′ to 3′ anchor regions were the haplotypes deemed completed. Supplementary Figure S2 illustrates the process of anchoring molecules, linking polymorphic labels, and our quantitative cutoffs for confirming a haplotype.

Molecule coverage and data quality cutoffs for CMAP haplotype confirmation were generated as follows. Based on optical mapping data of the 9 independently confirmed genomes, we found that >  = 5 × coverage of anchor and segmental duplicon-overlapping labels enabled the unambiguous determination of separate haplotypes within a genome and separate duplicons within haplotypes. This cutoff was assuming false positive labeling rates of 3–5% and false negative labeling rates of 9–17%, which were the recommended data quality cutoffs from Bionano Genomics. Genomes with values outside any of the previous ranges were re-run.

Finalized assemblies were manually curated into LCR22A and LCR22D localized haplotypes in the Bionano Access software for visualization (https://bionanogenomics.com/support/software-downloads/). New unique haplotypes were added to the chromosome 22 reference map file used in post-assembly alignments. This alternative reference CMAP catalog was used in subsequent assemblies and alignments for expedient confirmation. All final, curated molecule alignments and NAHR events were imaged from Bionano Access and annotated (see Supplementary Fig. S4 online; molecule alignment images: https://github.com/stevenpastor/nahr-22q/tree/master/all_molecule_alignments_confirmation_images).

### Confirmation of NAHR mechanisms

NAHR between LCR22A and LCR22D was determined through the visualization of inherited LCR22 haplotypes in probands from parents-of-deletion-origin. Label polymorphisms and label distance discrepancies present in parent-of-deletion-origin haplotype maps compared to proband haplotype maps enabled the reduction of ambiguous recombination sites (see Supplementary Fig. S3 online). LCR22A haplotypes were nearly always heterozygous and label distributions variable enough to determine exact LCR22A haplotypes involved in NAHR. Bionano Access visualization software was used to perform the aforementioned operations by visually comparing maps.

### Sequence-based annotations of optical map data

Reference build 38 was used to annotate sequences contained within recombination loci as defined in optical maps. UCSC Genome Browser “Duplications of > 1000 Bases of Non-RepeatMasked Sequence” track, “RefSeq gene predictions from NCBI—Annotation Release NCBI Homo sapiens Annotation Release 109 (2018-03-29)” track, and the “All GENCODE annotations from V31 (Ensembl 97)” track were used to annotate segmental duplications, genes, and pseudogenes, respectively^25,26^.

NCBI BLASTn^27^ was used to map translocation breakpoint sequences on 22q11: Type A (TBTA, accession: AB261997.1) to the human reference genome. Parameters used in the alignment included the removal of the low-complexity region and species-specific repeat filters to include repetitive sequence matches.

### Statistical testing

LCR22D inversion presence/absence was analyzed by using 2 × 2 contingency tables for the comparative groups. The comparison of LCR22A and LCR22D haplotype maps between parental groups was analyzed by using 2 × 32 and 2 × 5 contingency tables, respectively. All tests were defined as significant if p < 0.05. Fisher’s exact tests were performed on the 2 × 2 contingency tables using SciPy version 1.3.0′s “stats” module and the “stats.fisher_exact” function to determine p-values. The 2 × 32 and 2 × 5 contigency tables were analyzed using rpy2 version 2.94 with the “stats” package and the “stats.fisher_test” command, as the SciPy method can only be performed on 2 × 2 tables.

## Data availability

Cell lines used to map repeats are available upon request. Assembled genome map data and raw molecules have been deposited to the NCBI BioProject database (https://www.ncbi.nlm.nih.gov/bioproject/) under the accession: PRJNA640411. The custom scripts used in the study are available from https://github.com/stevenpastor/nahr-22q.

## Supplementary information

Supplementary file1 (PDF 7171 kb)
