# Supplementary material for: Optical mapping of the 22q11.2DS region reveals complex repeat structures and preferred locations for non-allelic homologous recombination (NAHR)
Source: Sci Rep. 2020 Jul 22;10:12235. doi: 10.1038/s41598-020-69134-4 (PMC7376033; doi:10.1038/s41598-020-69134-4)
Supplement: Supplementary file 1 — Supplementary file1 (PDF 7171 kb) [file 41598_2020_69134_MOESM1_ESM.pdf]

Supplementary figures for

Optical Mapping of the 22q11.2DS Region Reveals Complex Repeat Structures and Preferred Locations for Non-Allelic Homologous Recombination (NAHR)

Steven Pastor (PASTORS@email.chop.edu),[1]* Oanh Tran (TRANO@EMAIL.CHOP.EDU),[2] Andrea Jin (andreajin89@gmail.com),[2] Danielle Carrado (CARRADOD@EMAIL.CHOP.EDU),[2] Benjamin A. Silva (bsilva@pennmedicine.upenn.edu),[2] Lahari Uppuluri (lu42@drexel.edu),[4] Heba Z. Abid (hza23@drexel.edu),[4] Eleanor Young (eay25@glink.drexel.edu),[4] T. Blaine Crowley (CROWLEYT@EMAIL.CHOP.EDU),[2] Alice G. Bailey (BAILEYA1@email.chop.edu),[2] Daniel E. McGinn (MCGINNDE@EMAIL.CHOP.EDU),[2] Donna M. McDonald-McGinn (MCGINN@email.chop.edu),[2,3] Elaine H. Zackai (ZACKAI@EMAIL.CHOP.EDU),[2,3] Michael Xie (XIEM1@email.chop.edu),[1] Deanne Taylor (TAYLORDM@email.chop.edu),[1] Bernice E. Morrow (Bernice.Morrow@einstein.yu.edu),[6] Ming Xiao (mx44@drexel.edu),[4,5] Beverly S. Emanuel (EMANUEL@email.chop.edu)[2,3]

Supplementary Figures

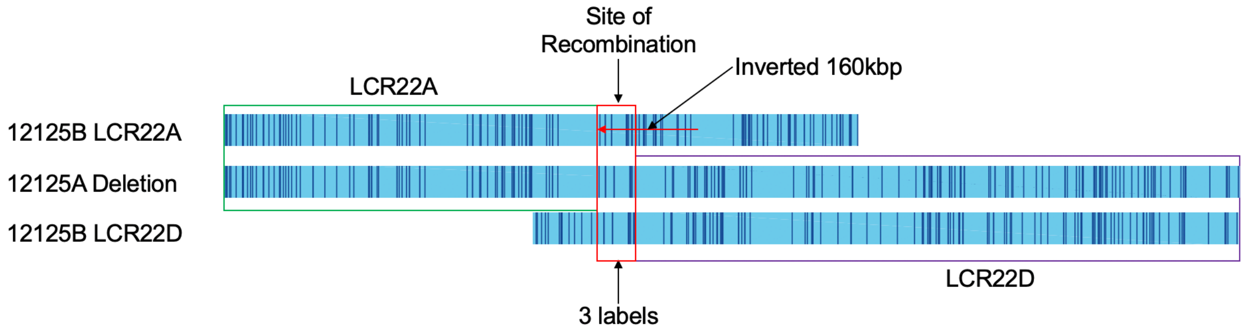

Supplementary Figure S1. Trio 12125 NAHR Mechanism with inverted 160kbp. Trio 12125 was the only family with a parent-of-deletion-origin consisting of a haplotype with an inverted 160kbp module (within LCR22A) as the site of recombination.

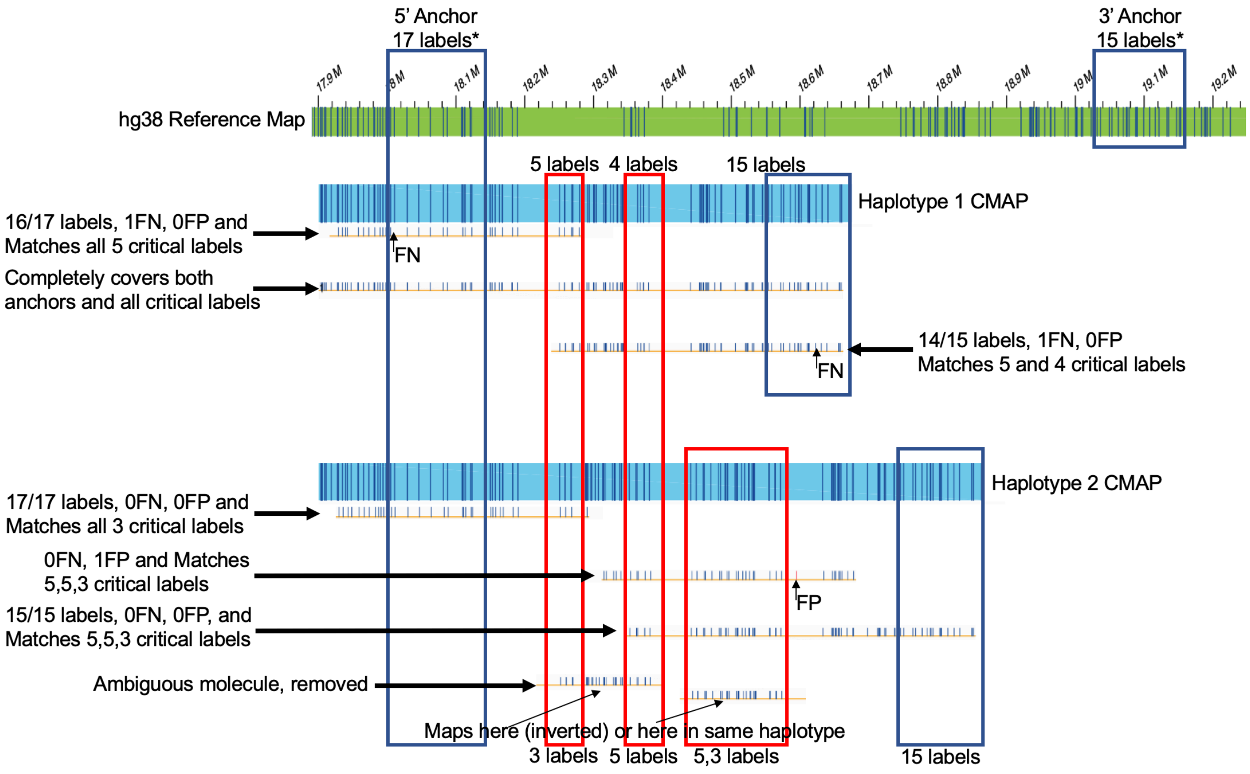

Supplementary Figure S2. Haplotype validation process. Two haplotype CMAPs (blue) mapping to the hg38 reference map (green) are anchored (blue box) and span segmental duplications (red boxes) with unique label patterns. Molecules (yellow) were used to confirm these 2 haplotype maps. All molecules mapping to anchor regions must conform to our cutoffs of a maximum of 1 false positive label and a maximum of 2 false negative labels. Comparing the 2 haplotype CMAPs indicates that the first labels in 160kbp modules after the 5' anchor are unique to their respective haplotypes. In other words, the 5'-most red box shows 5 labels in the 5' end of a reference-orientation 160kbp module in haplotype 1 whereas an inverted 160kbp module in haplotype 2 has 3 labels in the same mapped vicinity. Thus, anchored molecules which meet false positive and negative label specifications and match the respective haplotype map unique labels confirm each haplotype. The next unique set of labels is scanned and connected from the previous labels (second red box). Finally, the final unique label set is connected to the 3' anchor, again meeting the false positive and negative label specifications.

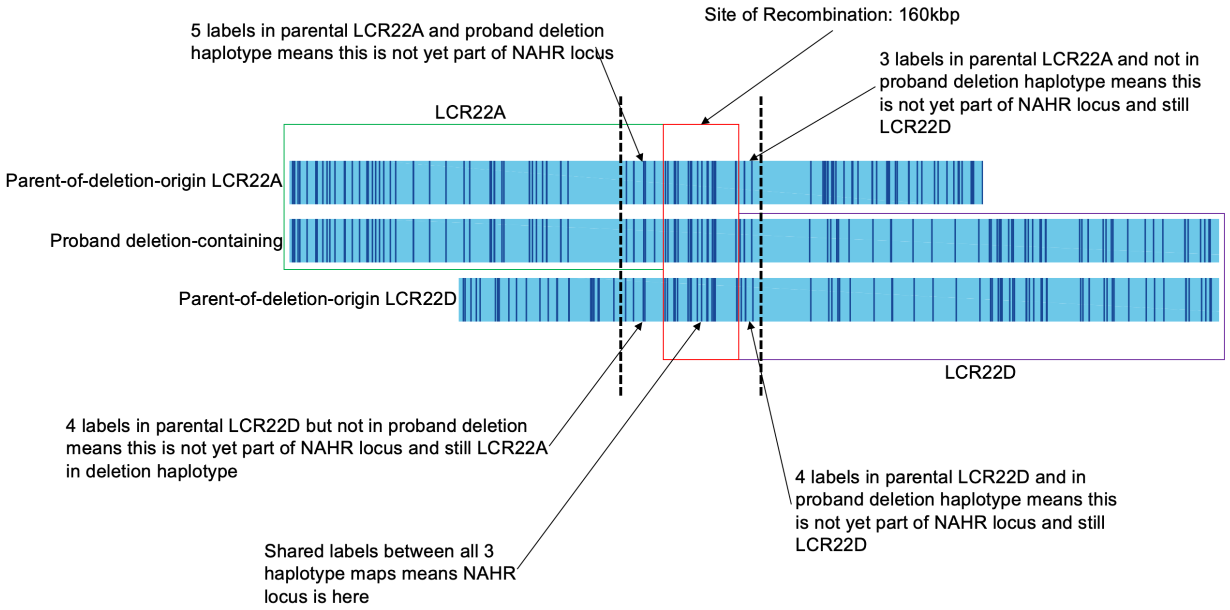

Supplementary Figure S3. NAHR locus reduction via polymorphic labels and differing distances between labels. Shared labels between a parent-of-deletion origin LCR22A haplotype map and a proband deletion-containing haplotype map indicate the regions before the exact NAHR locus. Previously, the entire 160kbp module was the ambiguous site of recombination (Demaerel et al. 2019). In our study, we used polymorphic labels to reduce this ambiguity from the entire 160kbp module to sections of it. Here, the 5 labels in the 160kbp module of the parent-of-deletion-origin LCR22A map shared in the proband deletion-containing haplotype map indicate that this ~40kbp section of the 160kbp module is not the NAHR locus. Comparing these labels to the parent-of-deletion-origin LCR22D haplotype map reveals 4 labels in the same locus, as compared to the reference map (not pictured). This implicates this section of the 160kbp module as LCR22A in the proband. Likewise, the 3 labels in the parent-of-deletion-origin LCR22A haplotype map compared to the 4 labels parent-of-deletion-origin LCR22D haplotype map reveal this ~20kbp section of the 160kbp module as LCR22D. Here, we reduced the ambiguous NAHR locus to ~100kbp. We have reduced other NAHR events down to <40kbp using this method.

11029 NAHR Mechanism

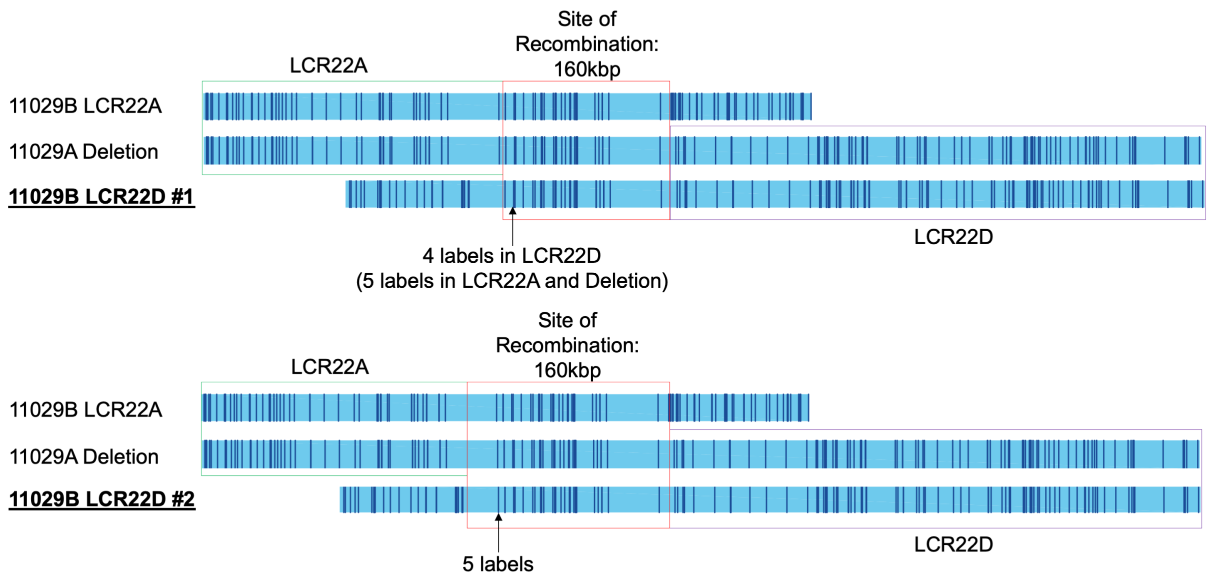

11041 NAHR Mechanism

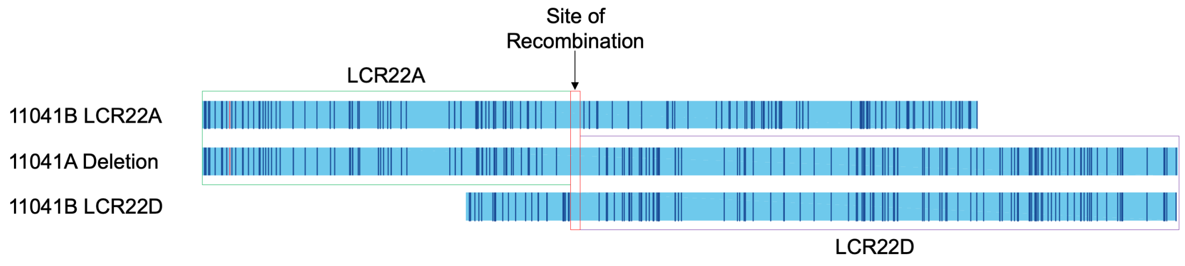

11163 NAHR Mechanism

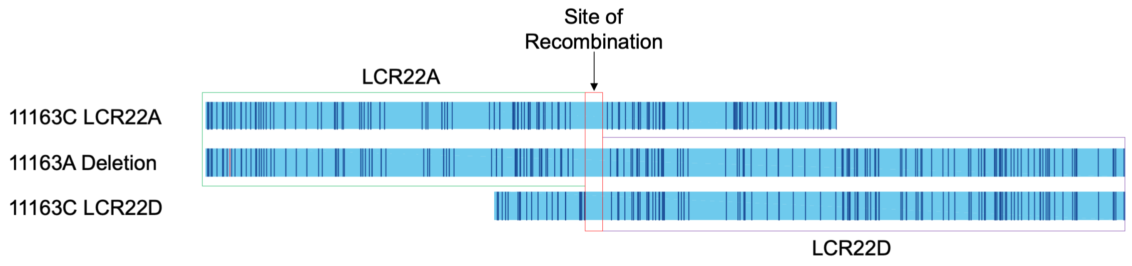

11175 NAHR Mechanism

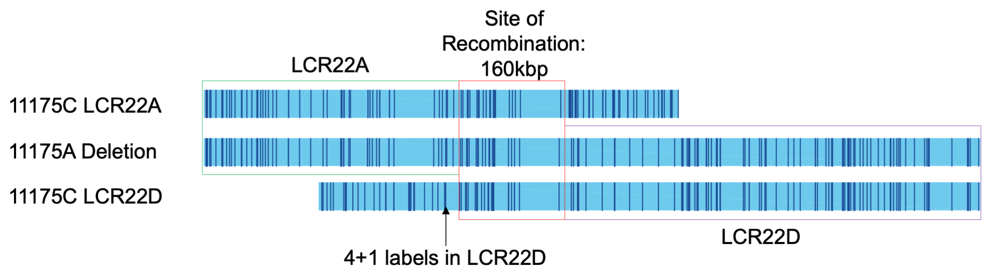

11186 NAHR Mechanism

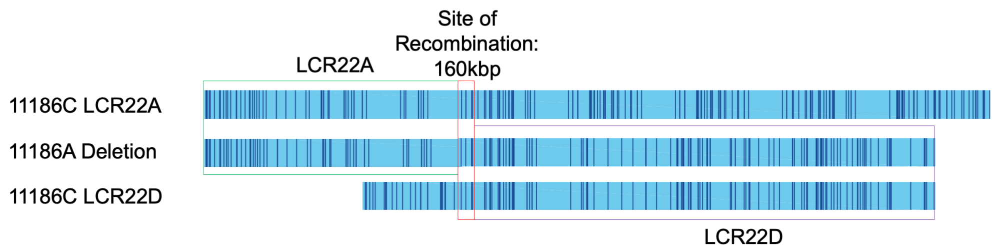

11263 NAHR Mechanism

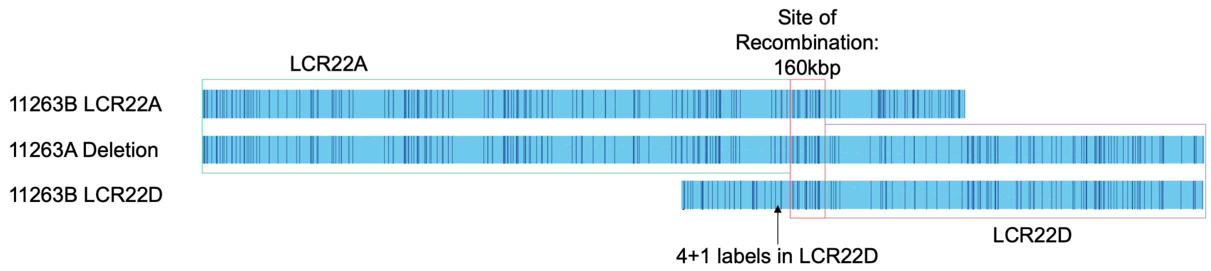

11269 NAHR Mechanism

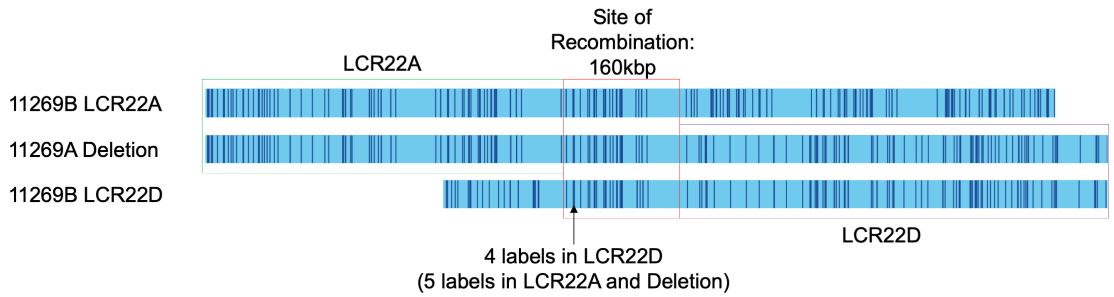

11276 NAHR Mechanism

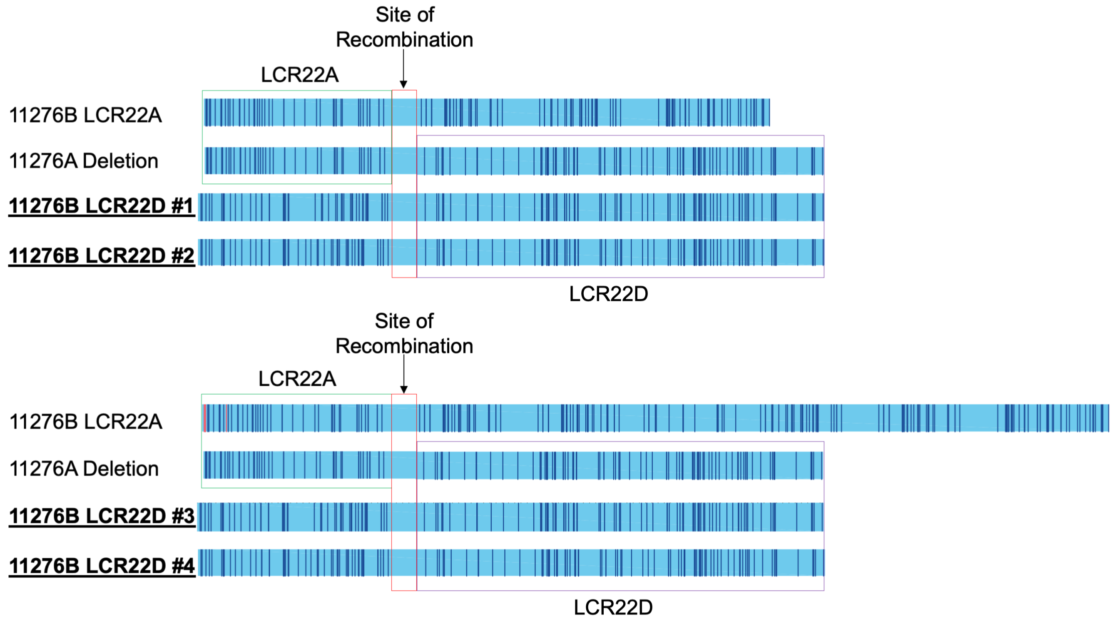

11280 NAHR Mechanism

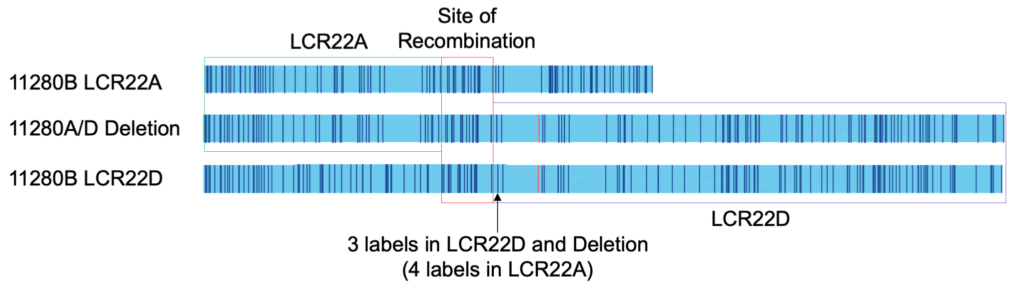

11345 NAHR Mechanism

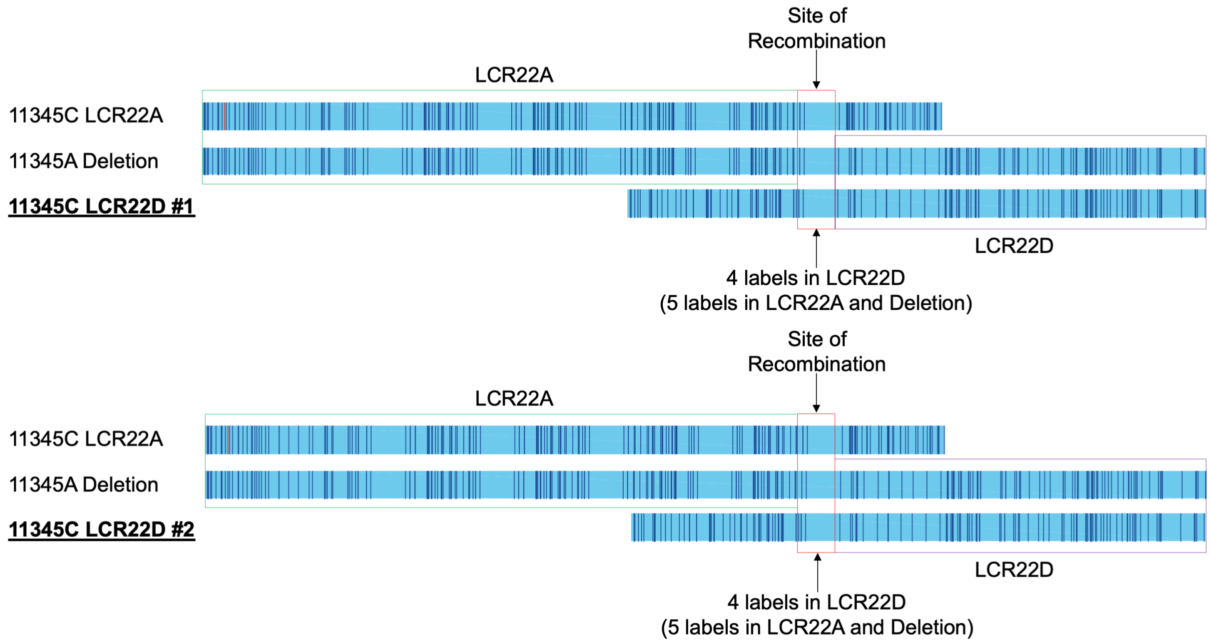

11418 NAHR Mechanism

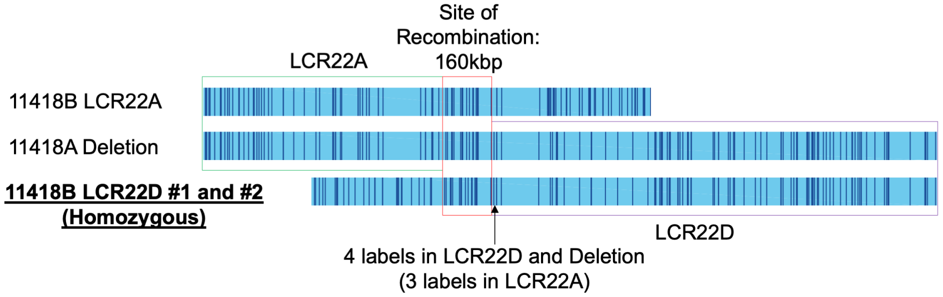

11434 NAHR Mechanism

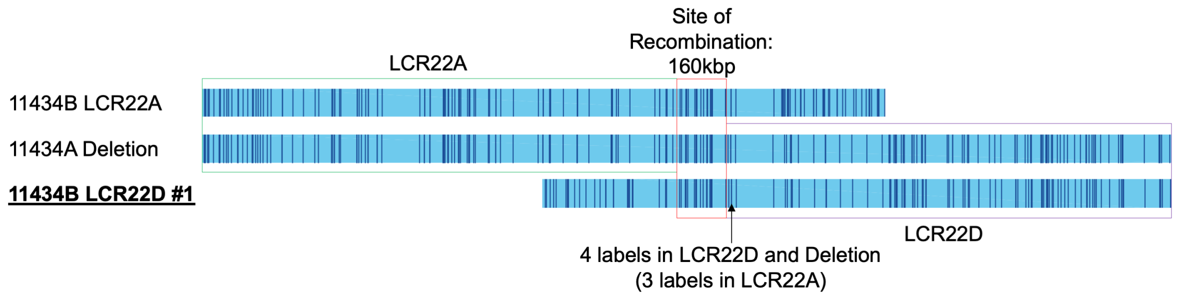

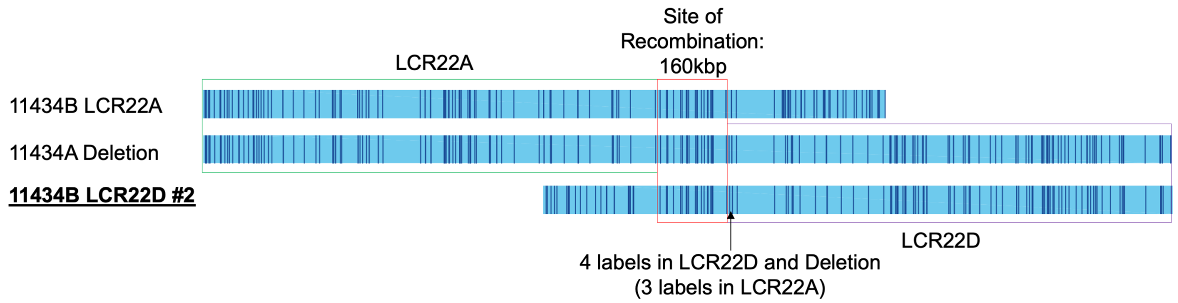

11465 NAHR Mechanism

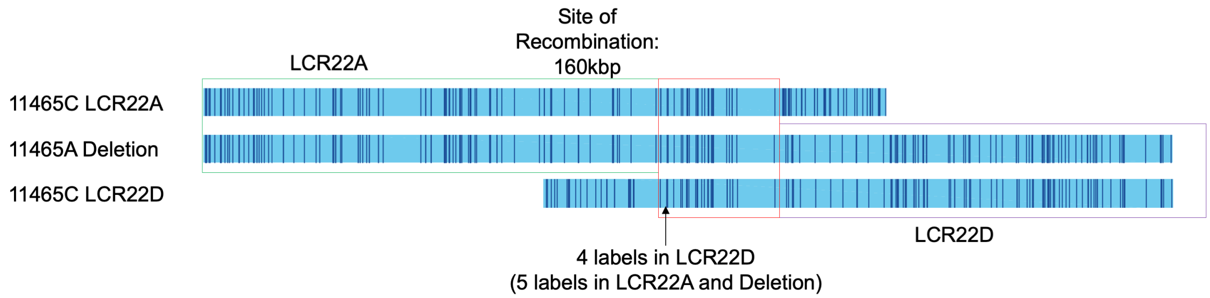

11668 NAHR Mechanism

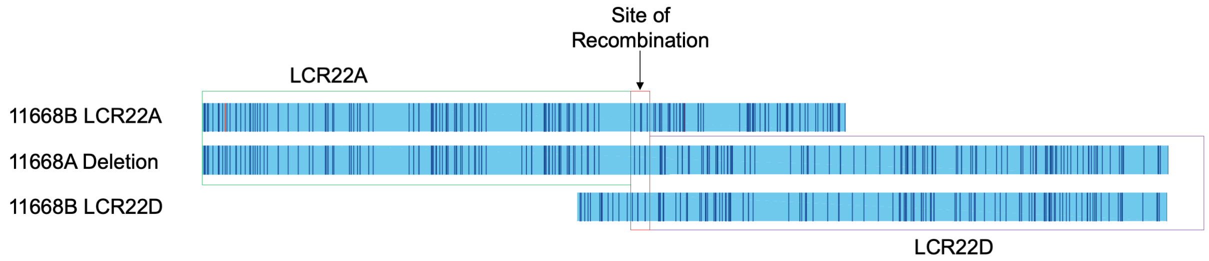

110733 NAHR Mechanism

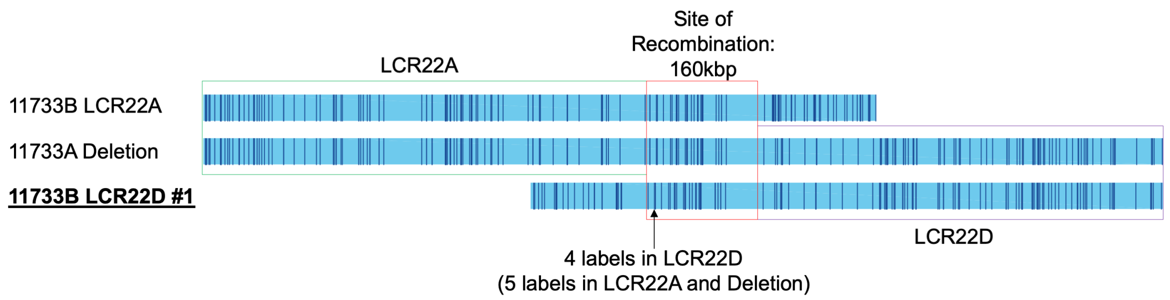

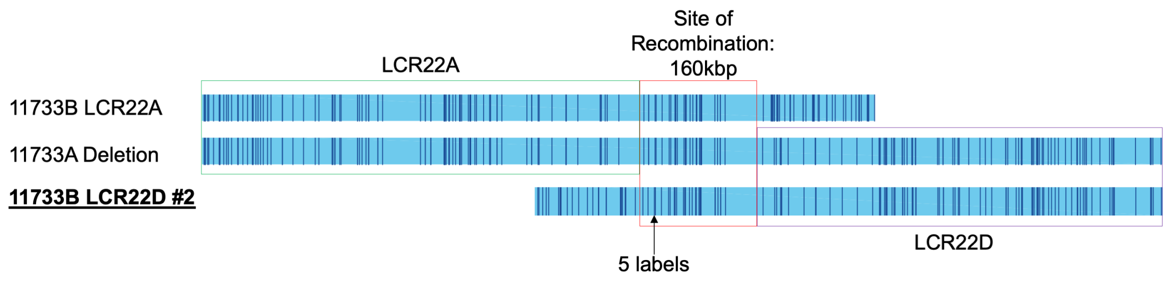

11740 NAHR Mechanism

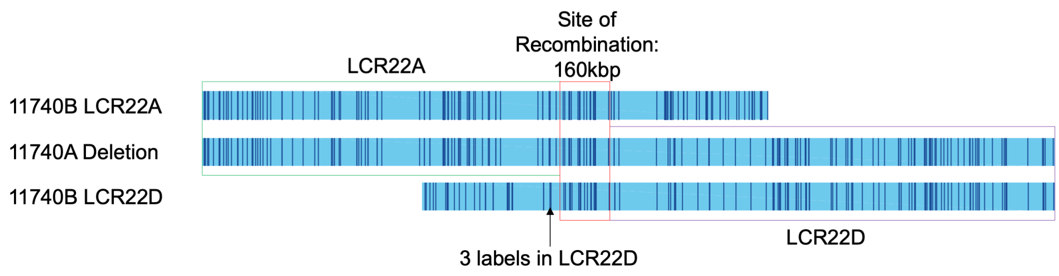

11744 NAHR Mechanism

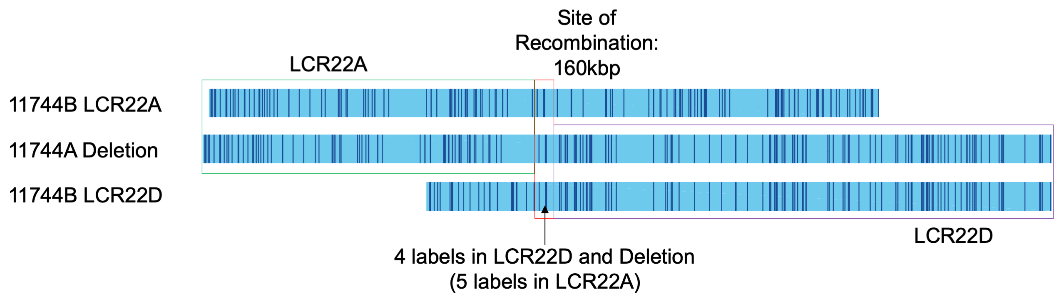

11766 NAHR Mechanism

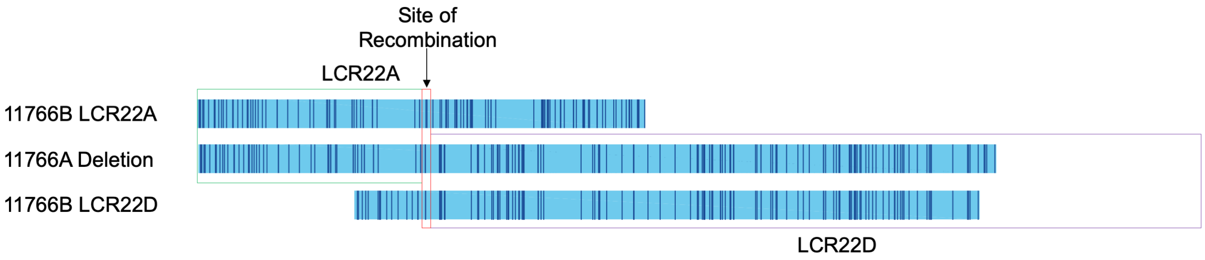

11800 NAHR Mechanism

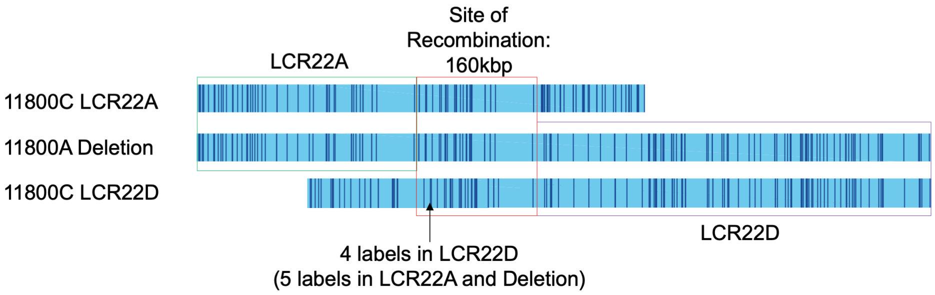

11922 NAHR Mechanism

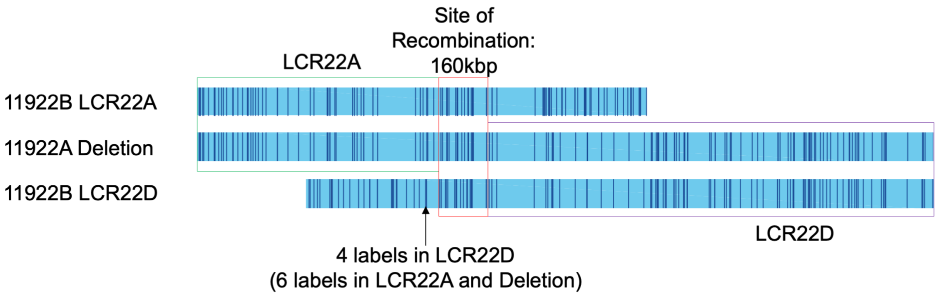

12018 NAHR Mechanism

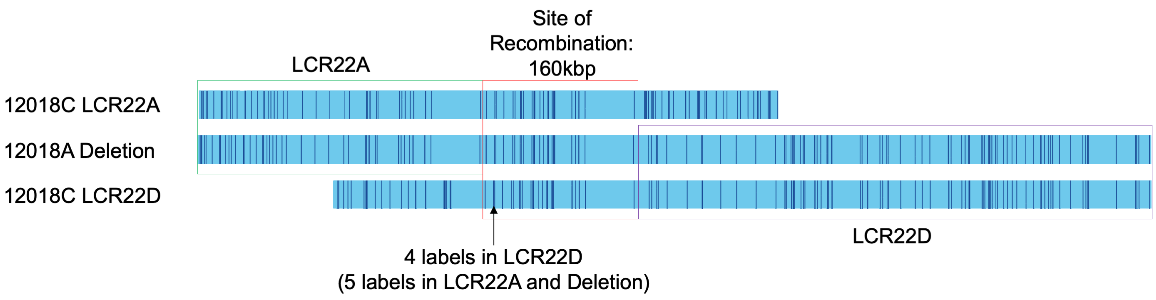

12125 NAHR Mechanism

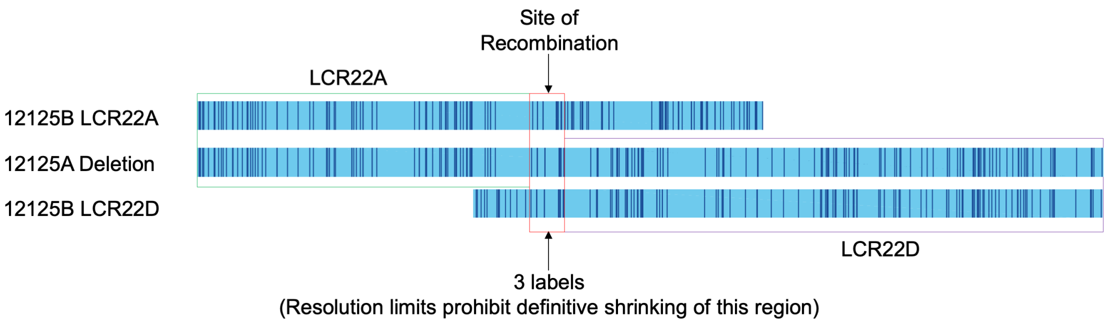

12459 NAHR Mechanism

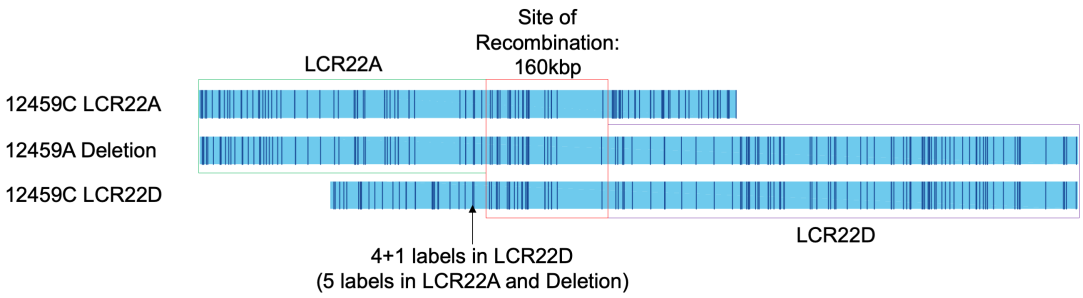

12468 NAHR Mechanism

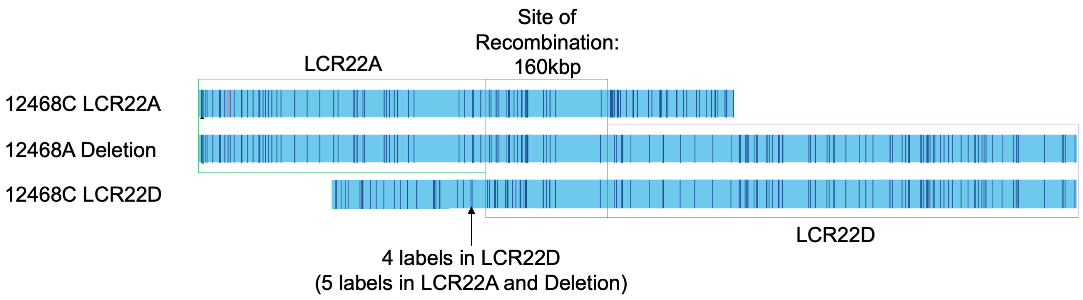

12486 NAHR Mechanism

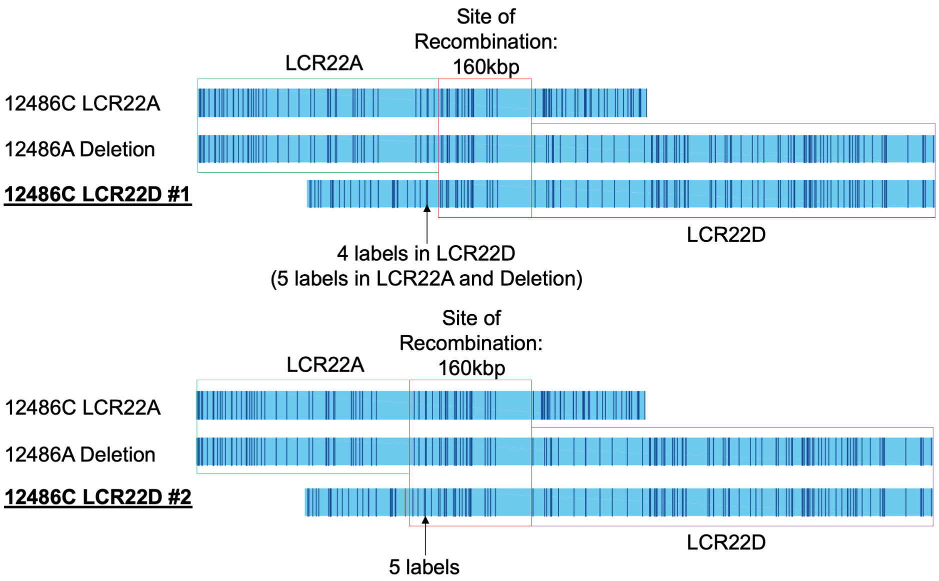

12523 NAHR Mechanism

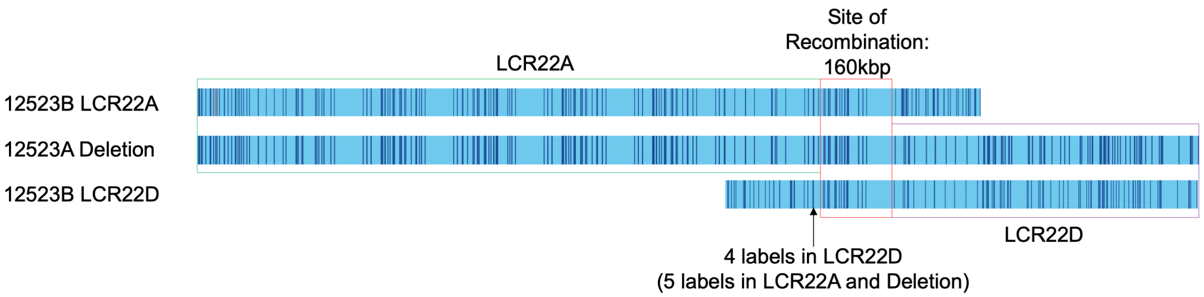

12533 NAHR Mechanism

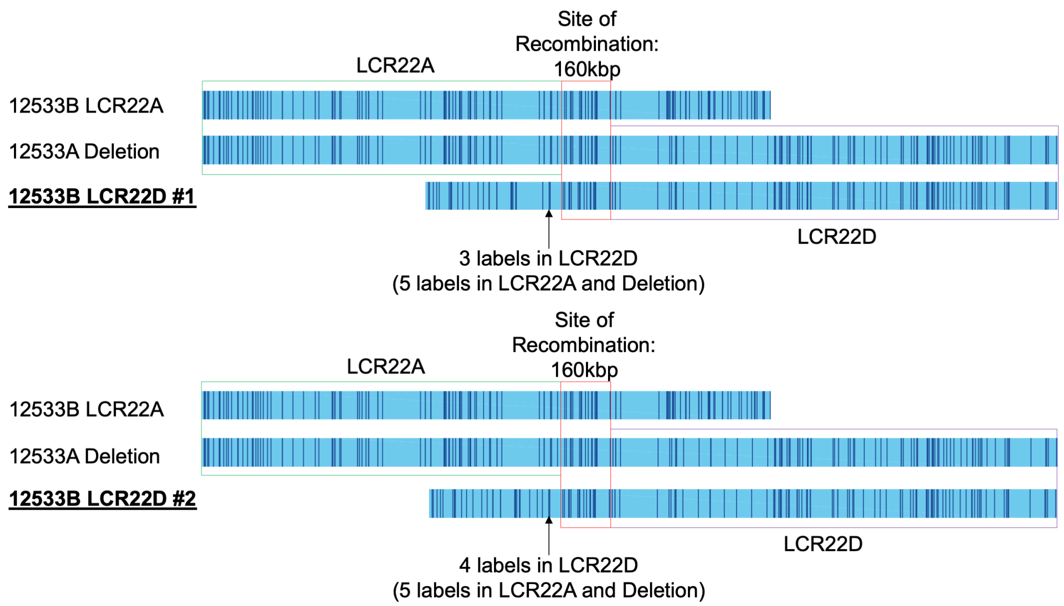

BM1452 NAHR Mechanism

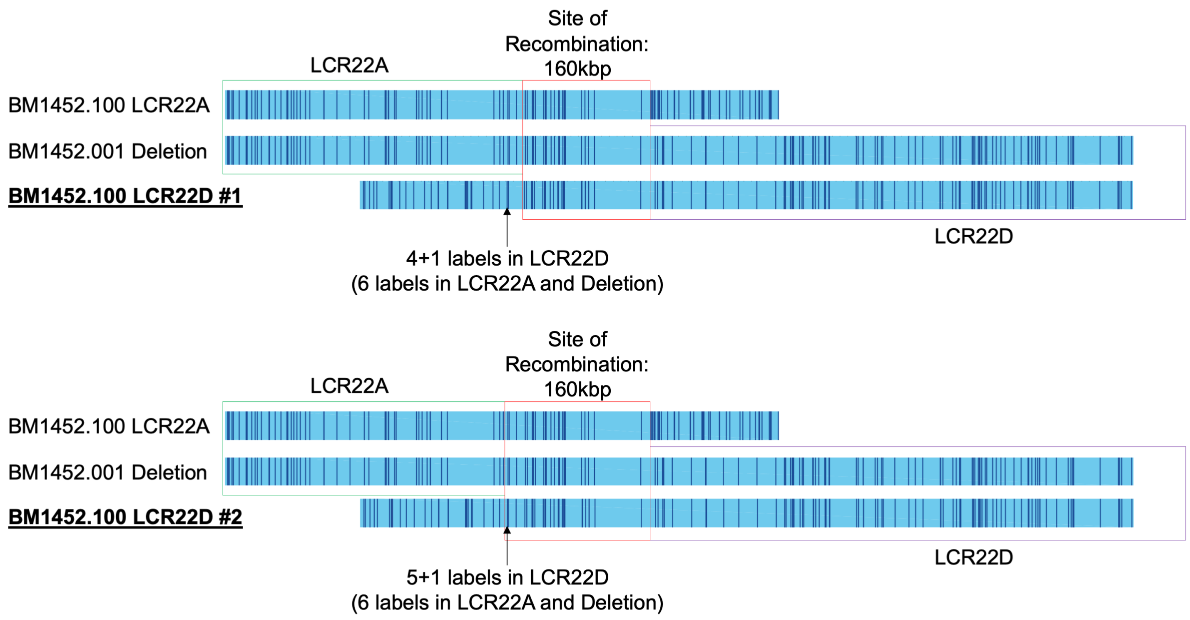

BM1453 NAHR Mechanism

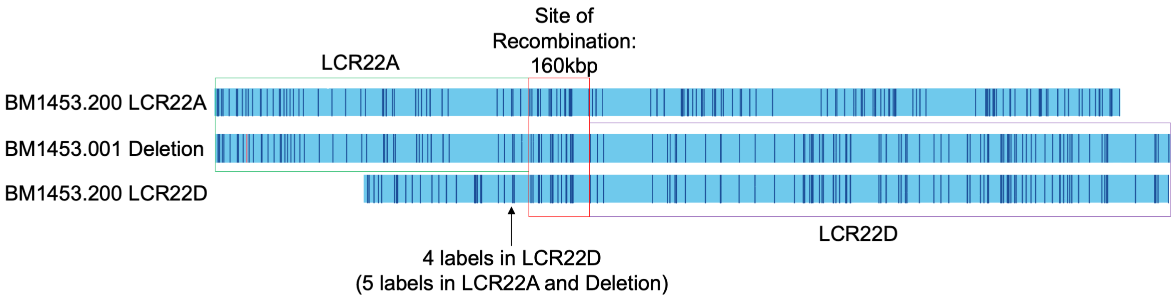

GM1038 NAHR Mechanism

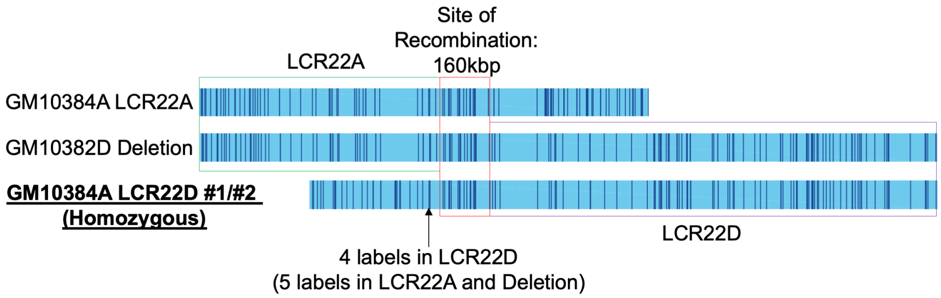

Supplementary Figure S4. All NAHR recombinations. The above figures are the 30 NAHR events. Each figure contains only the parent-of-deletion-origin's LCR22A (top map) and LCR22D (bottom map) contig haplotype maps participating in NAHR leading to the proband's deletion-containing haplotype map (middle map). In circumstances of ambiguity, the possible alternative event is presented as well.

Primer Name	Primer Sequence 5’ to 3’
22-19xAC-F	FAM-TGGTAGGGAGGGCACTAAGA
22-19xAC-R	CTGCCTCAGCCTCCTGAGTA
MS22C-F	HEX-CCTTTGCCCCCTGCACACCC
MS22C-R	TGGGGCTGCCAGAGCTGTCT
OT22A-F	HEX-TGCACATGTACCCCAGAACT
OT22A-R	CCCCATGCCTGAGTGACATA
OT22B-F	HEX-CTTGGGTTAGTGTGGGGTGA
OT22B-R	GCACATGTGACAAAGAGCCA
OT22D-F	FAM-TGCTTAACAGTTCTTCCCTCAA
OT22D-R	AATGACCTGAGATCACGCCA
D22S451b-F	HEX-CCCACATCTCCTCTTTTCCA
D22S451b-R	CAGGCCTTATTTGAGGTCCA
D22S1638-F	HEX-GACAACAGCAAATTGCACATT
D22S1638-R	TCACGCCACTACCCTCCAG
46STS-F	FAM-TCAAGTGTTGAAGGACATCTGG
46STS-R	TCCTGGGCATCTATCACACA
102STS-F	HEX-AGGATGCATAACAGGCTGCT

102STS-R	GAGGCCTTATGTGAGGTCCA
D22S941-F	FAM-CAGGTTACAAAGTACATTAACTT
D22S941-R	CAAGAAATGGTTGGAGCTGGT
115STS-F	HEX-AAAAAAAAAGCAACATCAAAGAGA
115STS-R	TCTAGGATTCCTTTCTGCTTAACA
D22S1648-F	HEX-CAGATGCTTCAGGAGAAGTG
D22S1648-R	AGTTGTCAGATGCCTAAGAGA
D22S264-F	TET-ATTAACTCATAAAGGAGCCC
D22S264-R	CACCCCACCAGAGGTATTCC
D22S264-2F	HEX-CAGGCAGGTCTTGAACTCCT
D22S264-2R	GCATTCCAATTACACTGCTGC
22-20xTG-F	HEX-AGCCTGAGAATTTCCCAAATCA
22-20xTG-R	CTGGGCGAGAGAGTGAGAC
22-26xAC-F	HEX-AGAGAGAGAGATCGGTTTTGCT
22-26xAC-R	CTGTGTCCAGCTTGTGTGTG
22-21xAC-F	HEX-AGCCAAAATCACGCCACTG
22-21xAC-R	AGAGATCATGGGTCCTTGCA
22-24xCA-F	HEX-ACAAAGCCTCTCAACATGACAG

22-24xCA-R	AGCCGAGTGTTTTGTGTGTG

Supplementary Table S1: Parent-of-origin deletion primers. Each row indicates the primer name and the sequence in which it binds, including any 5' modifications. The primers were used to determine parent-of-deletion-origin before optical mapping took place.
